# Past subarctic marine food web shifts recovered by sedaDNA and network analysis

**DOI:** 10.1038/s41598-026-60317-z

**Published:** 2026-07-12

**Authors:** Viktor Dinkel, Marc-Thorsten Hütt, Stella Z. Buchwald, Amelie N. Smith-Tønnessen, Ulrike Herzschuh, Kathleen R. Stoof-Leichsenring

**Affiliations:** 1 Polar Terrestrial Environmental Systems, Alfred Wegener Institute Helmholtz Centre for Polar and Marine Research, Potsdam, 14473 Germany; 2 Computational Systems Biology, Constructor University, Bremen, 28759 Germany; 3 Institute of Environmental Science and Geography, University of Potsdam, Potsdam-Golm, 14469 Germany; 4 Institute of Biology and Biochemistry, University of Potsdam, Potsdam-Golm, 14469 Germany; 5 Present Address: Interactions Hôtes Pathogènes Environnements (IHPE), Univ. Montpellier, CNRS, IFREMER, Univ. Perpignan Via Domitia, Montpellier, 34090 France

**Keywords:** Marine food web, Network analysis, Paleoecology, Sedimentary ancient DNA, Subarctic ecosystems, Climate sciences, Ecology, Ecology, Ocean sciences

## Abstract

Subarctic marine ecosystems are highly sensitive to current climate and sea-ice change but long-term evidence for food web shifts is lacking. Sedimentary ancient DNA (sedaDNA) data covering the last 124,000 years combined with network analysis provides evidence for a shift from glacial (GLC) bottom-up to a deglacial-interglacial (IG) top-down food web structure. A consensus network approach, calibrated on generalized Lotka-Volterra simulations, delivers distinct modules that potentially resemble interglacial and glacial trophic structures. The glacial food web with a dominance of sea-ice adapted primary producers, densely linked with each other, supports a high module robustness and resource-driven bottom-up control. In contrast, the deglacial-interglacial food web with predominant consumers, like salmon, herring and small whales, are trophically diverse but rather fragile. Our study assumes that mobile consumers are facilitated by habitat expansion through submerged shelves and ice-free conditions during deglacial-interglacial periods altering the composition and structure of higher trophic levels. Our study implies that future warming, sea-ice decline and sea level rise may affect the structure and stability of subarctic consumer-driven, top-down food webs with adverse consequences for ecosystem services.

**Supplementary Information:**

The online version contains supplementary material available at 10.1038/s41598-026-60317-z.

## Introduction

Current global warming is causing increased sea-surface temperature and sea-ice loss, affecting the composition of the Bering Sea ecosystem with consequences for ecological interaction and ecosystem functioning^1,2^. Generally, marine food web structures are strongly impacted by warming through poleward range shifts, enhancing algal blooms and nutrient availability^3,4^, which can affect predator-prey interaction, competition and resources availability^5,6^. In recent decades, a major ecosystem shift in the Bering Sea is observed through declining sea-ice, oxygen uptake and rising sea water temperatures assuming large effects on the Bering Sea food web^7^. Conceptual models^8^ of this oceanic region explain how decadal climate variability drives food web regulation dynamics indicating shifts from bottom-up (resource-driven) to top-down (consumer-driven) regulated food webs, while as a complement to both, wasp-waist control^9,10^ in which mid-trophic levels (secondary consumers) may exert control on their prey and predators, respectively, are known from North Pacific regions as well^11^. Although such decadal fluctuations are known, it remains unclear whether shifts in food webs and their regulation pattern are observable on geological timescales and across different climate periods. In particular the Beringia area was influenced by natural climate variation through glacial and interglacial periods leading to variation in sea level through changes in the volume of northern hemisphere ice sheets, that contributed to the exposure or submergence of the Beringia Strait^12^. Such interacting effects of climate, sea-ice, and habitat variability in the geological past likely have had coupled long-term consequences on the ecosystem functioning including changes in the composition^13^ and stability of trophic interactions^14^ and energy transfer^15^. However, information about past food web structures is rare and often restricted to taxa specific dynamics. For example, changes in terrestrial food webs of animals from the Quaternary are based on fossil isotopic data^16,17^ and environmental DNA (eDNA) from coprolites^18^, but also spatio-temporal co-occurrence data combined with current diet preferences are used to infer past trophic links^19^. Further, the dynamics of marine food webs under environmental change can be addressed with ecosystem modeling introducing trophic interactions and functional diversity in regional marine ecosystem models. Such studies, especially from polar marine ecosystems suggest temperature and salinity being the most important factors for the food web^20^ predicting a strong decline in fish biomass with warming^21^.

Another source for inferring past food webs are marine sediment cores that serve as paleo-archives across geological timescale, offering insights into long-term environmental history, including climatic fluctuations^22^ and biodiversity dynamics^23,24^. Paleorecords from the Bering Sea show low biological productivity^25^ due to greater seasonal sea ice^26^ during cold glacial periods. The loss of sea ice during deglaciation transformed the ecosystem from sea-ice adapted to an ice-free one with major shifts in the primary producer community, which has been shown by microfossil and geochemical data^12,27^. However, the consequences of a shifted primary production community for consumers at higher trophic levels is limited due to missing paleo-proxies. Currently, shotgun sequencing of sedimentary ancient DNA (sedaDNA) from sediment archives is the only proxy that enables the recovery of past ecosystems, in the absence of visible fossils, in sufficient taxonomic breadth and resolution^28–30^, allowing composition and potential ecological interactions including food web structure to be inferred. SedaDNA analysis from the Bering Sea has already revealed plankton community composition changes^31^ and whole pelagic and benthic ecosystem shifts and their relationship with temperature and sea-ice variability^32^ providing a profound understanding of the Bering Sea ecosystem through sedaDNA. However, an analysis of ecological interactions for the recovery of potential past food web structures based on sedaDNA is still lacking.

Ecological networks have become a key approach for analyzing complex food web interactions, particularly in modern ecosystems^33^. These network-based analyses help to quantify structural properties such as network modularity, connectivity^34^ and describe trophic interactions and their robustness^35–37^. Network inference methods are employed to identify causal and co-occurring relationships among taxa^38,39^, enabling to explore trophic interactions under environmental change^40^. However, achieving high predictive accuracy remains a significant challenge^41^ as it depends on the data quality, quantity and taxonomic resolution. SedaDNA shotgun data is typically sparse with a low number of samples and a coarse time resolution, which is challenging for network analysis. However, it is the only current method, which provides high taxonomic breadth and resolution enabling the investigation of whole ecosystem relationships. Therefore, studying sedaDNA and millennial-scale interactions necessitates the development of an appropriate network inference framework, along with robust methods for network analysis and validation. One essential method to improve the network prediction for sparse data is combining different inference methods into an ensemble as introduced in the DREAM^42^(Dialogue on Reverse Engineering Assessment and Methods) challenge in the context of gene regulation. The network ensemble approach increases the reliability of inferred links; however, distinguishing true trophic interactions from environmentally driven associations among taxa remains challenging.

This study applies a combination of network ensemble approach, which in the following we will call *consensus network* (CN), and database validation to infer potential trophic networks derived from sedaDNA data focusing mostly pelagic interactions. The sedaDNA network contains mixed associations (direct interactions and environmental relationships), which enable it to define highly connected modules that depict ecologically dependent communities across major trophic levels. Such modules potentially illustrate food webs under different environmental conditions^43^. Further, the connectivity^44^ within the network modules can be used to assess the robustness of the potential food webs, as fewer trophic links represent a high taxonomic dependency which is susceptible to perturbation and vice versa. Considering sedaDNA derived networks as potential food webs, flow metrics, known from theoretical approaches, can indicate the developmental state (juvenile or mature) of a food web. Metrics such as ascendency (A), development capacity (DC), and relative ascendency (A/DC) quantify ecosystem structure and organization, providing insight into food web maturity and stability. However, it is unclear if such as food web theory are applicable to a sedaDNA-derived food webs as the nature of the data only partly reflect the biomass of the detected taxa.

In this study, 42 samples from a marine sediment core from the Shirshov Ridge in the Bering Sea are used to infer a consensus network to reconstruct food webs and their long-term changes across glacial and deglacial-interglacial conditions over the past 124,000 years. We applied a comprehensive ecological network analysis on abundance data derived from shotgun sedaDNA. For that, we used a set of state-of-the-art methods: SPIEC-EASI^45^, Propr^46^, ESABO^47^, SparCC^48^, CCREPE^49^, and EcoCopula^50^ together with standard correlation analysis (Spearman). Our selection encompasses methods (e.g., SPIEC-EASI, EcoCopula) tailored for inferring direct species interactions^50,51^ and others (e.g., Spearman, CCREPE) that are more sensitive to indirect (environmental) and spurious^52^ interactions (Table S1). Intersecting the ensemble of inferences with a base network provides the ecological consensus network (CN), which contains direct and indirect interactions that are both theoretically possible and empirically observed. We validated this novel consensus approach with synthetic data using a generalized Lotka–Volterra model, evaluated the functional capabilities by applying modularity, robustness and flow metrics, and confirmed direct and indirect trophic links with the GloBI^53^ database. Our approach constitutes an initial attempt to merge network methodology and food web theory with sedaDNA time series data to investigate shifts in food web structure and fragility across geological time scales, providing a proof-of-concept based on well-established methods, yet requiring further development and validation.

## Material & methods

### Sample material and data processing

The SO201-2-77KL core was recovered in 2009 during the Sonne cruise SO201-KALMAR leg 2. The site location is the Bering Sea Shirshov Ridge (56.3305°N, 170.6997°E) at a depth of 2135 m. The corresponding geochemical study^12^ states that linear sedimentation rates (LSR) and bulk accumulation rates (AR_Bulk_) on Shirshov Ridge range from 11 to 16 cm kyr⁻¹ (7–15 g cm⁻² kyr⁻¹), enabling millennial scale reconstructions. Generally, LSR and AR_Bulk_ were higher during colder intervals and peaked during Termination I (20–10 ka BP). Past marine productivity was reconstructed using multiple proxies (total organic carbon, CaCO₃, biogenic opal, biogenic barium, and XRF logging). Productivity remained low during cold periods and high during warm stages, with maxima during interglacials. The age model was established using high-resolution core logging data (color b*, XRF scanning) and various proxies, with the sequence derived from graphic correlation between core 85KL color b* data and Dansgaard–Oeschger (DO) climate variability recorded in the δ¹⁸O NGRIP record. Further information about the sediment core was described in the corresponding geochemical studies^12,27^.

The sediment core was stored at Geomar, Kiel, Germany in a dedicated cooling chamber. The clean subsampling of the core was conducted in a core sampling lab with full body suits and sterile materials to prevent contamination of the sediment DNA with modern DNA. Based on the existing age model^12,27^, samples were selected to cover the full temporal range of the sediment core (1.82–123.86 ka BP). However, periods of climatic transitions (end of the Eemian and beginning of the glacial period; the deglaciation; the beginning of the Holocene) were sampled in higher temporal resolution than the glacial period to capture community changes during these rapid climatic changes. More details about the sample preparation and processing in the paleogenetic laboratories at Alfred Wegener Institute (AWI) Helmholtz Center for Polar and Marine Research in Potsdam Germany can be assessed in Buchwald et al.^31^. Briefly, the DNA extraction was done on clay- to silt-sized siliciclastic material with the DNeasy PowerSoil Max Kit (Qiagen, Germany), followed by a purification of extracts with GeneJET PCR Purification Kit (Thermo Scientific, USA) and a single-stranded DNA library preparation according to Gansauge and Meyer^54,55^ with an input material of 30 ng DNA. Indexed and pooled libraries were sequenced on a NextSeq2000 Sequencing device (Illumina) in paired-end mode (2 × 100 bp).

Raw sequencing data was quality checked with Fastqc v. 0.11.9^56^, deduplicated with clumpify (BBMap package v. 38.87), trimmed and merged with fastp v. 0.20.5^57^, and taxonomically classified with Kraken2^58^ using a confidence threshold of 0.2 (default 0.0) against the nt database (release April 2021). Kraken2 classifies reads based on exact matches of constituent k-mers (k = 35 bp; minimizer length = 31 bp) to sequences in the reference database. Each matching minimizer is assigned to the lowest common ancestor (LCA) of all reference sequences containing that minimizer. Taxonomic assignments are subsequently weighted by the cumulative evidence provided by matching k-mers across a read. The confidence threshold specifies the minimum fraction of k-mer evidence that must support a taxonomic assignment; thus, a threshold of 0.2 requires at least 20% of the total k-mer evidence to be consistent with the assigned taxon. Increasing the confidence threshold reduces potentially spurious classifications at the expense of a lower proportion of classified reads.

The classified reads were filtered using a curated list of 297 marine families. The list originates from publications^31,32^ on the marine environment of this region but has been further curated, excluding families that are endemic to the Southern hemisphere. We limited our analyses to family level, because assignments for genus and species level are less confident due to large database gaps for marine species^59,60^. Post-mortem damage patterns were checked for selected families using Map Damage v 2.0.8^61^ and C/T ratios at the 5’ end of the DNA reads were plotted for the sediment core samples merged to six age groups (Supplementary file S1).

### Characterisation of taxonomic families and trophic levels

A curated list of 297 marine families was used to filter the shotgun data. Non-photosynthetic bacteria, archaea and viruses were not selected as the study focuses on the pelagic ecosystem. The aggregation of the taxonomic compositional data on family level is advantageous because of higher read counts per family (as species and genera are included) and lower bias introduced by database limitations compared to lower taxonomic identifications. Further, the family classification is sufficient for inferring major trophic levels and trophic interactions^62^. However, grouping species into a family clade may generalize highly diverse species, overlook their unique or opposing functions, and group different age classes within a species that can occupy varying trophic levels, which generalize the trophic structures and interaction in the underlying dataset.

Selected families were classified into 19 taxonomic categories and collapsed in four major trophic levels (1-primary producer, 2-primary consumer, 3-secondary consumer, 4-tertiary consumer) (Supplementary Table S2 & S3) using an automated screening of public databases using R. Firstly, families are taxonomically verified by the “World Register of Marine Species” (WoRMS) database^63^. For each family the script queries PubMed using broad trophic-related keywords (e.g. “trophic level”, “trophic position”, “diet”, “feed*”, “feeding”, “feeding ecology”, “food web”,”autotroph”, “heterotroph”, “primary producer”, “photosynthesis”, “‘predat*”, “herbivor*”, “omnivor*”, “mixotroph”) combined with the family name and its genera (retrieved dynamically from the WoRMS taxonomy service). Queries are chunked to avoid PubMed query length limits, and results are downloaded in batches using the NCBI Entrez API. For each article, DOI, title, abstract, journal, year, authors, and keywords are extracted and written to a CSV file. From the listed literature we manually extracted the trophic level classification for the families. Families that were not found in the PubMed database were looked up manually and reference links from web sources are provided.

Mean trophic levels (MTLs) are computed for a set of families (e.g., within a module). MTL is based on the number of families, where families are counted for each trophic level (1 to 4) and then normalized to return fractions for each trophic level in a module. The sum of fractions multiplied by their respective trophic level comprises the MTL of the module.

The proxy for climatic conditions is the δ¹⁸O NGRIP dataset^64^, where age data points are selected closest to the sample ages. Then the extracted NGRIP δ¹⁸O-isotope values are centered and standardized by subtracting their mean value and division by standard deviation. This results in the δ¹⁸O fluctuations between glacial and interglacial conditions and for simplicity it is called temperature in this study (Supplementary file S1).

Each family’s relative abundance is Spearman correlated to the δ¹⁸O NGRIP temperature with the R function corr.test^65^. The thresholds are |0.3| for correlation strength and p-value < = 0.05. The correlation coefficient of |0.3| is intentionally moderate, as our goal is to capture general trends in families’ climate preferences indicated by δ¹⁸O NGRIP data, rather than to establish strong or causal links to the δ¹⁸O NGRIP proxy itself.

### Network inference

**Fig. 1:**
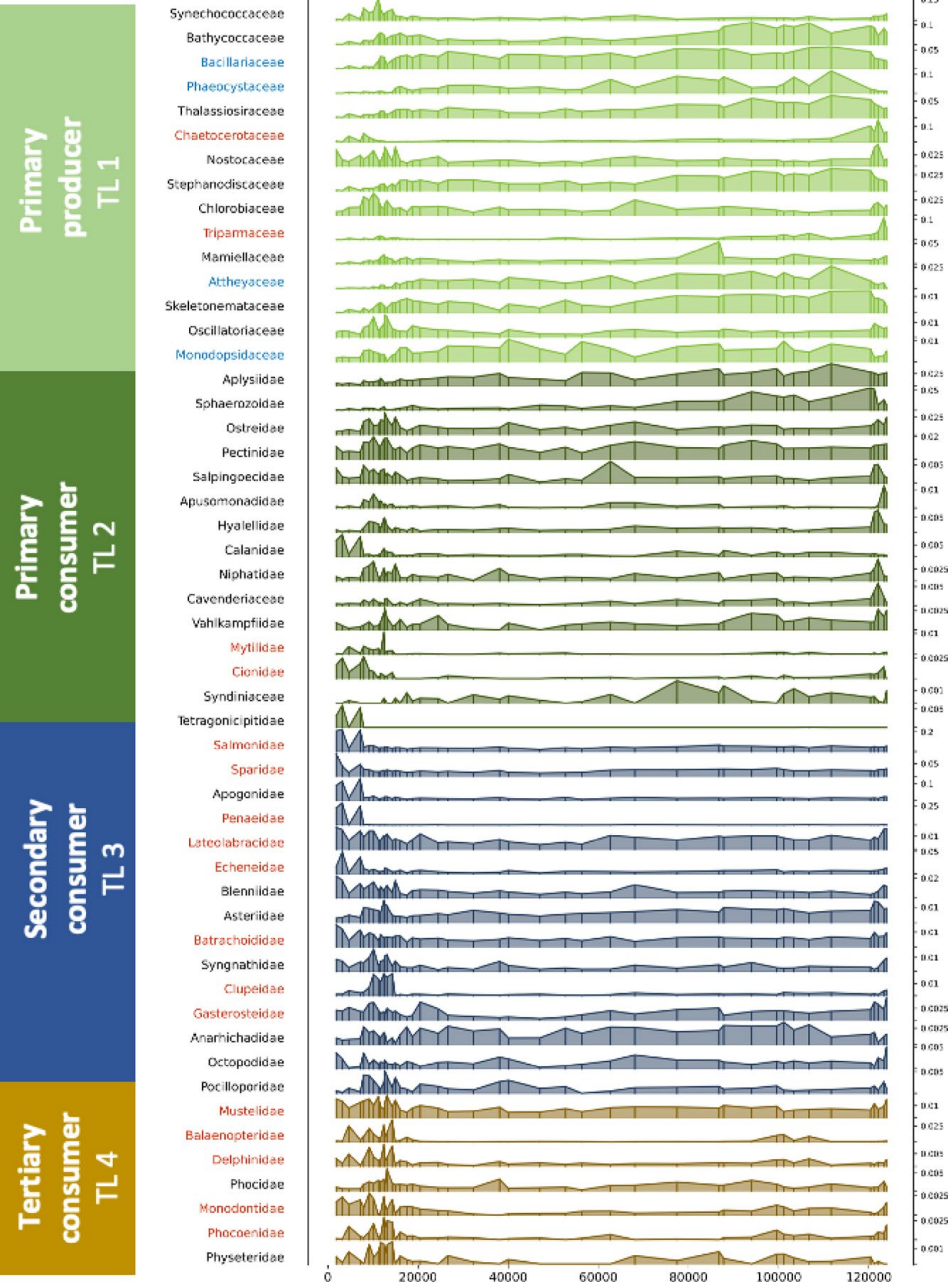
SedaDNA from the core SO201-2-77KL reveals dynamics in marine biodiversity (family level) over the last 124,000 years. The variation in relative abundance is displayed for up to 15 of the most prevalent families sorted into four trophic levels. Families that are significantly (|R| >= 0.3 and p <= 0.05) correlated with the paleo temperature (derived from the δ¹⁸O NGRIP record64) are highlighted in red (positively correlated) or blue (negatively correlated). Vertical lines show the respective sample ages.

For the purpose of network inference of ecological systems, we selected a diverse set of methods—SPIEC-EASI, Spearman, Propr, ESABO, SparCC, CCREPE, and EcoCopula—that are novel or frequently employed in ecological network analysis. These methods represent a selection from state-of-the-art tools and were chosen to encompass a methodological range of both direct and indirect interactions (Table S1). Some methods, such as SPIEC-EASI and EcoCopula, are particularly tailored for inferring species interactions, while others, like Spearman and CCREPE, may capture broader environmental influences on the network. This selection is neither exhaustive nor intended to identify the optimal approach but serves as a representative snapshot to explore the methodological diversity and capture the multifaceted nature of ecological networks, Fig. 1.

**Fig. 2:**
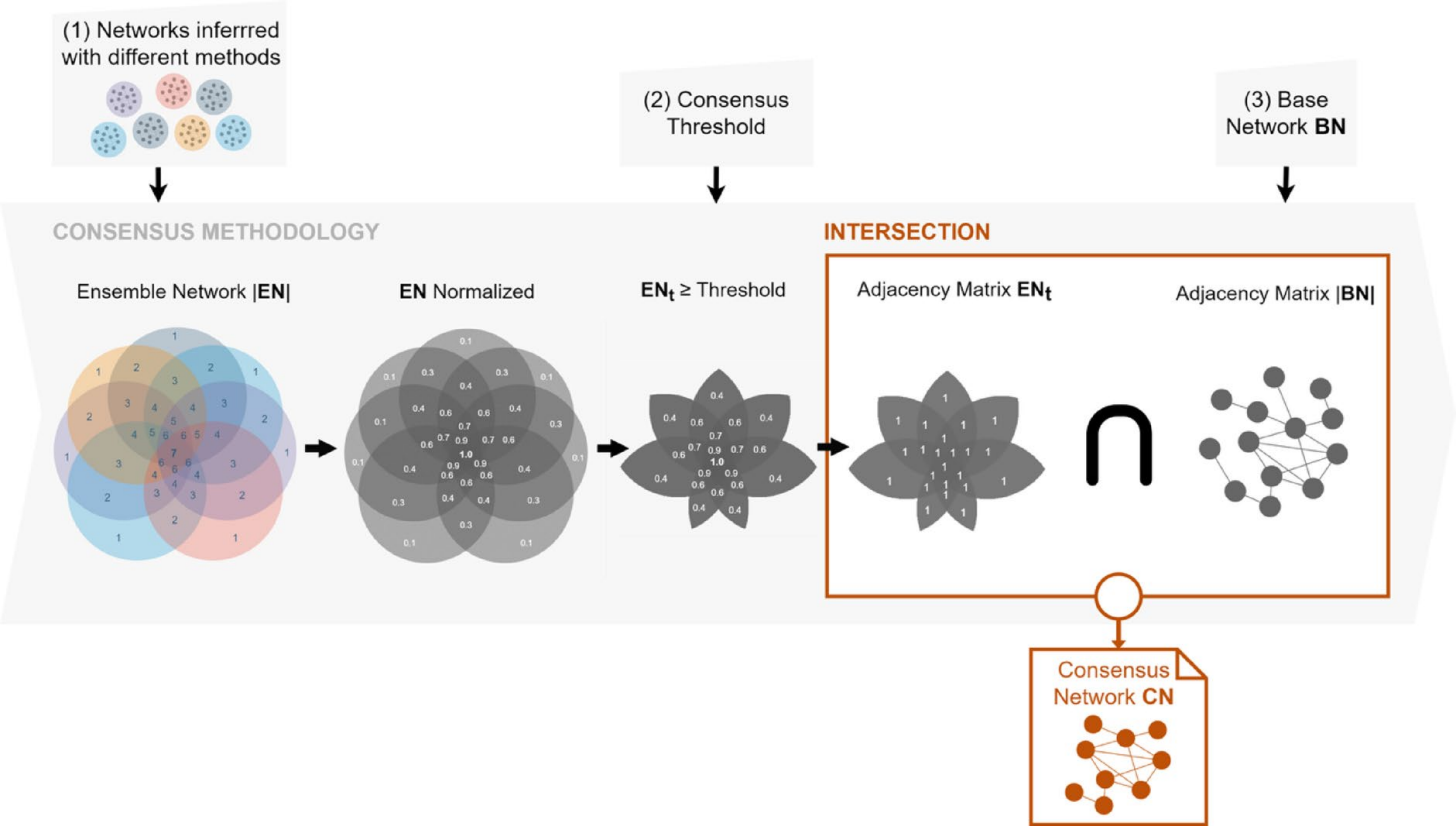
Consensus methodology combines networks from multiple inference methods to validate a base network. Three inputs are required: (1) a set of inferred networks for the ensemble network, (2) a consensus threshold parameter between 0.0 and 1.0, and (3) a base network, which in intersection with the ensemble network, produces the consensus network. Each network is represented as a symmetric, unsigned adjacency matrix. These matrices are aggregated to create the ensemble network (EN) of mixed association types. EN is then normalized to [0.0, 1.0] based on the number of inference methods used, filtered by a specified consensus threshold, and transformed to an adjacency matrix resulting in the ensemble network ENt. The threshold defines density and consensus strength of the network (in this study a threshold of 4/7 was used). Finally, the consensus network (CN) is formed by validating the intersection of the base network BN and ENt. This step is the deviation from a general ensemble approach, as the ensemble network EN is only used for validation and sparsification of a target network BN.

The networks are inferred using the R package NetComi and method netConstruct^51^, R package ecoCopula^50^, and python implementation of ESABO^47^. The settings for network inference were identical, where applicable. Relative abundances (total sum scaling) were used for network inference. Families were not filtered based on minimum counts or occurrences in samples. For ESABO, relative abundances were transformed to a binary presence-absence matrix, as required by the method. EcoCopula used the statistical model *stackedsdm* with unnormalized raw read counts to avoid incompatible fractions. Using NetComi, the sparsification method was set individually for each inference method, depending on applicability. In the resulting networks “nodes” represent the families and “edges” the links between the nodes. The sparsification thresholds were constant and empirically established to return an edge number in the same range for all methods, specifically to return a sparse network as well as having more edges than nodes (around 2,000 edges, ca. 5% of a complete network given 297 families). For methods outside NetComi like ESABO and ecoCopula, their specific threshold parameters (z-score = 1.3, lambda = 0.5) were also empirically established. Each inferred network was transformed to return an unweighted, unsigned, symmetrical adjacency matrix with diagonal set to 0 (Fig. 2).

**Fig. 3:**
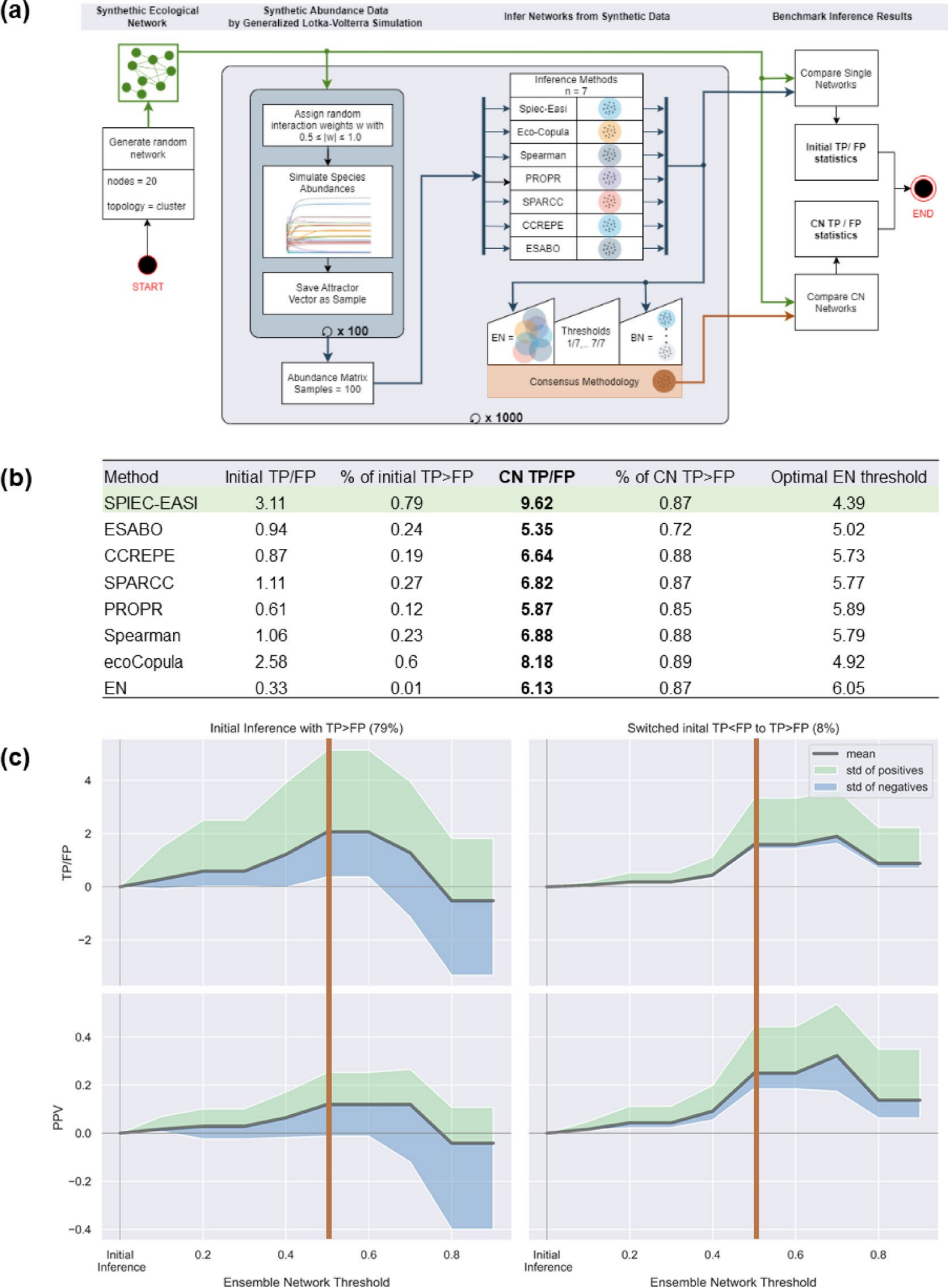
Benchmarking and calibration of inference methods on synthetic data shows increased precision with consensus methodology. (**a**) Illustrates the experimental setup for 1000 experiments where a synthetic ecological network is used to generate synthetic abundance data using the generalized Lotka-Volterra model. Networks are inferred from this synthetic data with different inference methods, including the consensus method. The benchmark compares the original synthetic ecological network with networks from individual inference methods as well as the consensus network to assess each inference precision as a TP/FP ratio and positive predictive value (PPV). (**b**) Shows the benchmarked TP/FP ratios of networks from individual inference methods as well as their improvement through consensus sparsification by ensemble network EN. (**c**) Detailed inspection of SPIEC-EASI’s TP/FP ratio and positive predictive value (precision) with increasing EN threshold (relative to its initial value). With increasing EN threshold, the mean TP/FP-ratio and precision increases compared to the initial value until a threshold of approximately 0.6. The deviation towards improvement (green) appears more pronounced than towards declining results (blue).

We employed SPIEC-EASI as the primary inference tool, a compositionally aware method optimized for sparse compositional data^45^, such as sedaDNA. This method was chosen due to its demonstrated ability to identify direct interactions while minimizing spurious edges caused by compositional effects as described in the results and Fig. 3. However, recognizing the limitations of individual methods, we adopted a consensus approach to improve overall network accuracy. The consensus approach combines multiple inference methods, leveraging “crowd knowledge” to reduce false positive rates. While this strategy does not recover false negatives (undiscovered interactions), it enhances the reliability of detected edges.

The consensus approach refines a base network (SPIEC-EASI) using an ensemble network derived from seven inference methods as shown in Fig. 2. Each edge in the ensemble network is the normalized (weighted between 0 and 1) sum of the seven inference methods, representing the proportion of methods supporting it. A threshold of 4/7 is applied to retain only edges with weights above this value. The adjacency matrix of the filtered ensemble network is then intersected with the adjacency matrix of the SPIEC-EASI network, retaining only edges supported by both. This approach enhances network reliability by combining consensus from multiple inference methods with SPIEC-EASI’s structure.

### Simulation of synthetic data

The inference quality of each method was quantified with experimental benchmarks based on synthetic data generation (Fig. 3a) and a Snakemake^66^ pipeline for workflow automation with multiple development environments. A random network serving as the template for the family interaction matrix was generated with *n* = 20 nodes (families) and an arbitrary clustered topology using the make_graph() function from the SPIEC-EASI R package. Importantly, SPIEC-EASI was used solely to generate the template graph structure (i.e., the adjacency pattern), without incorporating its precision or covariance properties, and without employing its built-in synthetic data generation. The actual abundance data were simulated independently using a generalized Lotka–Volterra model on the family interaction matrix, generated from the template network via the following modifications. Each of the undirected edges is assigned a random interaction strength between [0.5, 1.0] with a random sign. In the family interaction network, each of the undirected edges (family-family interaction) is randomly set with values between − 1 and + 1, while also ensuring that each value has an absolute magnitude of at least 0.5 to represent strong interactions. This approach ensures that the interaction matrix reflects the predefined network structure (random cluster) and emphasizes stronger interactions. The resulting matrix includes a mix of symmetric and asymmetric interaction types, such as predation, competition, and symbiosis, but excludes asymmetric edges where only one of two species has an interaction strength of 0. This network serves as the true interaction matrix *A* between nodes which is used for synthetic data generation. The diagonal of *A* is set to 1. Abundance dynamics for each species i were simulated using a generalized Lotka–Volterra model (see Eq. (1)). Numerical integration was performed in Python using the odeint() function from the SciPy integrate module (scipy.integrate). It involves the interaction matrix *A* and uniform random species growth rates *r*_*i*_ ∈ [0.01, 1.0], carrying capacities *k*_*i*_ ∈ [1, 100] as well as initial abundances *x0*_*i*_ ∈ [10, 100]. Each simulation of species abundances over 100 timepoints resulted in a final attractor state of abundances, which facilitates one sample of an experiment. Each experiment consists of 100 samples with each resulting from a new set of random vectors *r*,*k* and *x0* and the constant interaction matrix *A*. The 100 samples of attractor states constitute the abundance matrix *M* of each experiment. This has been repeated until data from 1000 experiments was accumulated.

1 $$\:\frac{d{x}_{i}}{dt}\:=\:{r}_{i}{x}_{i}\:\left(\:1-\:\frac{Ax}{{k}_{i}}\right)$$

EcoCopulas lambda was set to 0.55 and ESABOs z-score threshold to 1.3. Each experiment results in seven inferred networks to a total of 7000 networks. All inferred networks are compared to the true (ecological) interaction matrix *A* to get each initial inference quality (Fig. 3b). The number of true positives and false positives is measured for each inferred network to get the TP/FP-ratio and precision (positive predictive value) numbers. Then, for each experiment, the consensus network was generated (Fig. 2) and applied to single networks. The outcome was binned into two categories, either an improvement of TP/FP > 1 was achieved or not (Fig. 3c). Simulations without improvements are treated separately (excluded) because their randomized parametrization setups display computational artifacts. More specifically, the simulations included multiple tests of each randomized interaction matrix, with simple checks to exclude pathological cases (e.g., species abundances exploding exponentially above a threshold or all collapsing to zero). When such checks failed, the randomized interaction matrix was re-initialized with new properties until the simulation ran smoothly. Nevertheless, we assume that some simulation-setups passed the tests but remained unstable or ecologically unplausible, leading to poor inferences. Therefore, only those simulations in which species interactions were generally detectable were retained.

### Modules and distinct communities

Modules within the network were identified using the Python function greedy_modularity_communities() from the networkx.algorithms.community module with a resolution parameter of 1.0, corresponding to the standard modularity definition without bias toward smaller or larger communities. The algorithm iteratively merges nodes and communities to maximize modularity, thereby grouping nodes that are more strongly connected to each other than expected under a random network with the same degree distribution. Further, we defined modules as distinct modules where at least one family is correlated to δ¹⁸O NGRIP-temperature while no other family is present with opposite δ¹⁸O NGRIP-correlation in the same module.

### Globi interactions

The ecological consensus network is formed by the overlap of different inference methods, highlighting the diverse interaction types captured by each edge and resulting in their classification as mixed associations. These interactions are validated, using the GloBI database^53^, to confirm their ecological consistency with known trophic relationships (Supplementary file S1). Therefore, we compared all consensus network (CN) edges to known trophic relationships in the GloBI database. A null model, based on 1,000 randomized networks using double edge swaps, was used to estimate the expected overlap by chance.

### Ecosystem maturity

The maturity of an ecosystem is parametrized by multiple factors (flow metrics) based on information theory according to Ulanowicz^67^ comprising total system throughput *TST*, complexity *H*, average mutual information *AMI*, ascendency *A*, development capacity *DC*, and relative ascendency *A*/*DC*. For testing the potential of flow metrics, we considered the sedaDNA consensus network as a potential food web to explore the network’s potential for ecosystem functioning via the flows and the system’s structural organization. Time series data on flow metrics, evaluating ecosystem maturity (relative ascendency A/DC) through measures such as flow efficiency (ascendency A) and development capacity (DC), provide quantitative understanding of both dietary specialization and the fragility of ecosystems.

For the computation of (directed) flows the undirected graph is transformed to a directed graph by replacing each undirected edge into one ingoing and one outgoing edge. In Ulanowicz’s Ascendancy Theory of Ecosystem Development, edges may be represented either qualitatively (as unit flows) or quantitatively, with edge weights corresponding to measurable quantities such as biomass. Assuming that the relative read counts of the families relate to biomass, each outgoing edge’s weight is its flow, where the weight is the relative abundance of the source node divided by its out-degree. This means the outflow of each taxonomic family is its relative abundance equally distributed to its outgoing neighbors. We assessed the developmental status of individual samples, considering each family’s relative abundance as its outflow and the sum of all flows within the system (either the LCC or a specific module in isolation) as a sample’s *TST*. For each sample, a simulation graph is constructed containing only nodes from the network and associated edges of families with relative abundance greater than 0. Calculating the maturity values for LCC and modules, their subgraphs are extracted. This means only the nodes within a subgraph are the occurring families and the edges between them. Hence, *TST* is the sum of relative abundances of each subgraph’s nodes. In this study, Eq. (2, 3, 4, 5 and 6) depend on the methodology to compute flow indices^68^ with *N* being the total number of nodes of a subgraph. *T*_*ij*_ is the weight of the edge from node *i* to *j*. *T*_*i*_ and *T*_*i*_ are the total relative abundances of nodes *i* and *j*, respectively, within a given sample.

2 $$\:AMI\:={\sum\:}_{i\:=\:1}^{N}{\sum\:}_{j=1}^{N}\left(\frac{{T}_{ij}}{TST}\right){\:log}_{2}\left(\frac{{T}_{ij}\:\:TST}{{T}_{i}{T}_{j}}\right)\:\:\:\:$$

3 $$\:{H}_{c}=\:-1{\sum\:}_{i\:=\:1}^{N}{\sum\:}_{j=1}^{N}\left(\frac{{T}_{ij}}{TST}\right){\:log}_{2}\left(\frac{{T}_{ij}^{2}\:}{{T}_{i}{T}_{j}}\right)\:\:\:$$

4 $$\:H\:=\:AMI\:+\:{H}_{c}$$

5 $$\:A\:=\:TST\:*\:AMI$$

6 $$\:DC\:=\:TST\:*\:H$$

For each sample, the flow metrics are extracted, leading to a time series of ecosystem development metrics. Each of the individual metric’s time series is Spearman correlated to a centered and standardized δ¹⁸O NGRIP temperature proxy, indicating significant climatic influence on a systems development.

The Red Sea relative sea level (RSL) provides a continuous, high-resolution relative sea-level reconstruction providing a global eustatic proxy that reflects the changes of ocean volume. Despite regional variations in gravitational pull and hydro-isostatic shelf flexing, the global eustatic signal isolated by Grant et al.^69^ accurately captures the overarching glacial-interglacial trends and magnitude of water volume changes required to reconstruct the first-order flooding history of the Bering Sea. A 1-kyr Gaussian filter was applied to smooth the high-resolution dataset from to the short-term hydrographic noise.

The flow analysis of IG1, separated by top-down, bottom-up and intraguild flows, is illustrated in Supplementary Fig. S6. The temporal A, DC, and A/DC values were clustered using the KMeans algorithm from the Python module sklearn.cluster, with the number of clusters set to two (n_clusters = 2).

### Robustness analysis

The robustness of the network is assessed by iterative removal of nodes (knockout extinction) with all connected edges and measuring the size of the largest connected component (LCC). This measures how many nodes are disconnected from the LCC after each deletion, giving the speed of disintegration. High robustness is associated with low disintegration speed and vice versa. The LCC is either the LCC of the consensus network (CN) or a module. Deletions always target the LCC. For the random targeted deletions, 1000 experiments were performed in which nodes were picked randomly within the LCC.

The critical path of the network’s LCC was identified using the Python function minimum_node_cut() from the NetworkX library. In modules, the critical path was established by constructing an extinction tree, where each possible knockout path was measured. Leaf nodes (terminal nodes in the extinction tree) at the lowest depth returned the critical path(s). If multiple critical paths were present at the same depth, a representative path was manually picked. After each deletion, the size of the LCC was assessed using the connected_components() function from the NetworkX library.

## Results

### SedaDNA derived composition-temperature relationships

The SO201-2-77KL core from the Shirshov Ridge is 11.78 m long, recovered from an elevation of −2,130 m and holds a record going back to 123,860 years BP. Each of the 42 shotgun-sequenced samples, averaging 43 million reads, underwent Kraken2 classification against the non-redundant nucleotide database (built in April 2021), resulting in 184 million taxonomically classified reads (3–21% classified reads per sample at a 0.2 confidence threshold), with 62.7 million at the family clade level. For filtering, a curated list of 297 marine families predominantly of pelagic origin (0.6% of classified reads, 353,884 reads), including pro- and eukaryotic primary producers and eukaryotic consumers, yielding 2,379 to 45,612 reads per sample before normalization to relative sample abundance. These families were sorted into 19 taxonomic categories corresponding to four major trophic levels (Supplementary Table S2 & S3). Primary producers (55%, 164 families) and secondary consumers (23%, 69 families) dominated. The remaining families were primary (19%, 55 families) and tertiary consumers (3%, 9 families). Figure 1 illustrates the relative abundances of major families (95% of the filtered dataset), their trophic levels, and temperature relationships in the sediment record.

We use δ¹⁸O from the North Greenland Ice Core Project^64^ (δ¹⁸O NGRIP) as a paleoclimate proxy, specifically as a reference for marine climate variability and cascading effects such as sea-ice dynamics, seasonality, and nutrient availability. This purpose is verified in Supplementary Fig. S1, which demonstrates a significant negative correlation between δ¹⁸O NGRIP and IP_25_, a sea-ice proxy, from both cores (77KL, the core of this study, and KL12, a neighboring core). A significant positive correlation between relative abundance of a family and δ¹⁸O NGRIP is found for 29 families (including Chaetocerotaceae, Salmonidae, and Balaenopteridae, see Fig. 1), indicating a preference for interglacial conditions, while 22 families (including Bacillariaceae, Phaeocystaceae, and Gadidae) show a negative correlation, preferring glacial conditions. Primary producers, including some diatoms (Attheyaceae) and haptophytes (Phaeocystaceae), decrease from 124 ka to the Holocene, while other diatoms (Chaetocerotaceae) and cyanobacteria (Synechoccocaceae) increase in warmer periods. DNA-abundance of primary consumers is underrepresented, contributing only 8% to the total sedaDNA. Sea squirts (Cionidae), jellyfish (Pelagiidae) and copepods are more prevalent in interglacials. Worms (Cirratulidae, Priapulidae) have higher abundances during glacial conditions. Secondary consumers, mostly herbivorous fish, like salmon (Salmonidae) and sea breams (Sparidae), predominate during interglacials, and predatory crustaceans (Penaeidae) during glacials. Tertiary consumers, including dolphins (Delphinidae), sea otters (Mustelidae), porpoises (Phocoinidae) and baleen whales (Balaenopteridae), have higher relative abundance during deglacial-interglacials, particularly in the Holocene.

### Ecological consensus network from sedaDNA family data

Ecological relationships between 297 families, mixed associations including direct interactions and environmental relationships, were inferred using the consensus methodology illustrated in Fig. 2. The consensus method was calibrated and validated by synthetic experiments as shown in Fig. 3a, b. These results show that the consensus approach yields consistent inference quality improvements, evident in the ratios of true positives (TP) to false positives (FP) using a consensus threshold of 0.5. The calibration through synthetic experiments resulted in setting the consensus threshold to 4/7 (between 0.5 and 0.6 as shown in Fig. 3c) and choosing SPIEC-EASI as the base network, because it shows the highest TP/FP ratio (9.62). SPIEC-EASI was also chosen because of its methodological emphasis on direct interactions and compositionality. Furthermore, results provide evidence that using an ensemble network (EN) itself as a solution is inadequate, as evidenced by lower end initial TP/FP ratios (0.33 average) and after a threshold was applied (6.13 average).

**Fig. 4:**
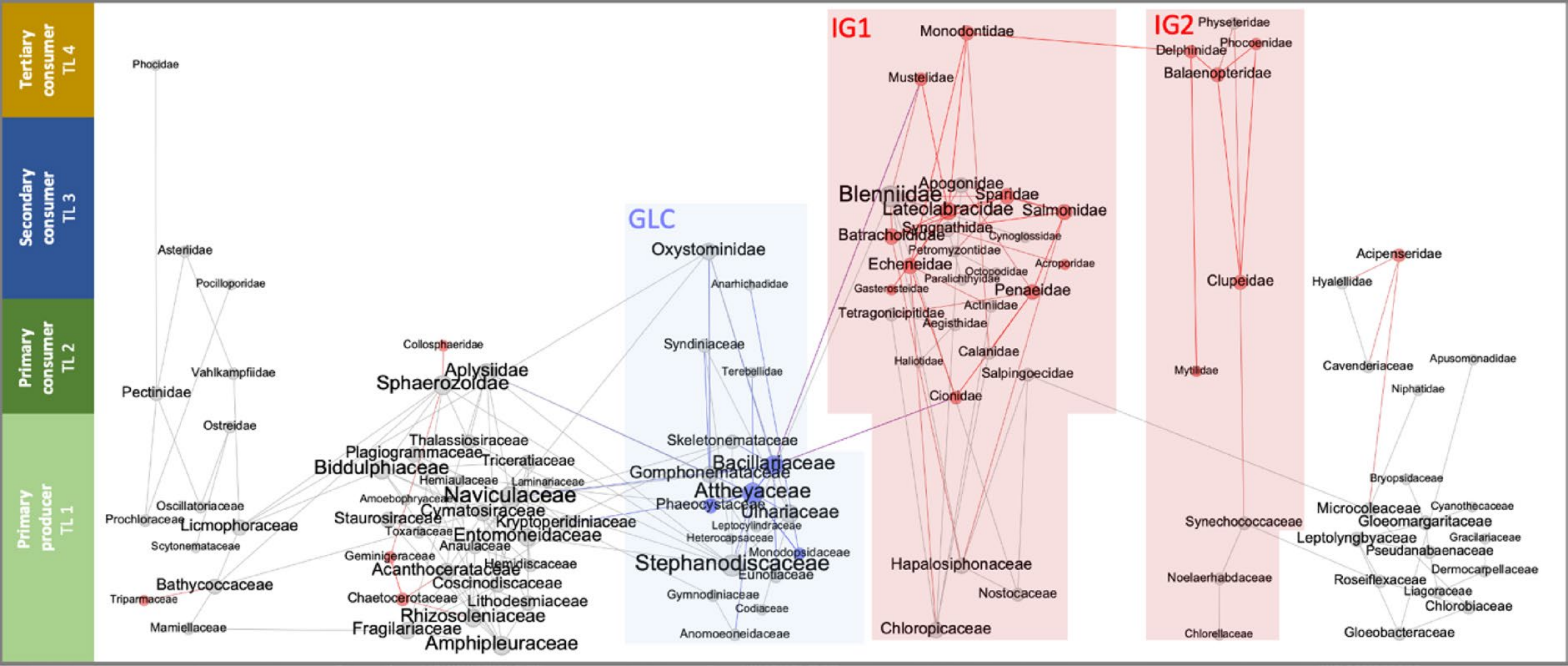
The Bering Sea network and its largest connected component (LCC) comprises six taxonomic modules across four trophic levels. Node size represents the node degree centrality. Red and blue nodes show positive and negative Spearman correlations, respectively, with temperature, see text in Figure 1. IG1 and IG2 are highlighted in red as they contain the most families positively correlated with temperature, while module GLC is highlighted in blue as it is the sole module within the LCC containing families with a negative correlation to temperature.

Applying the calibrated consensus method on sedaDNA data results in a consensus network (CN; Fig. 4) with 144 nodes (families) and 212 edges (links). Seven network inference methods were utilized, as depicted in Fig. 2, with the base network SPIEC-EASI (297 nodes, 976 edges) filtered by the ensemble network (EN) featuring 7990 edges. The resulting CN integrates shared edges across methods to enhance robustness in ecological association inference. SPIEC-EASI, as the base network, exhibits full overlap with the consensus, contributing 212 edges (100%). Other inference methods show varying degrees of overlap and contribution to the consensus network edge selection. EcoCopula (89%) and CCREPE (86%) exhibit the highest agreement with SPIEC-EASI, with 188 and 183 shared edges, respectively. Similarly, Spearman (86%) and SparCC (76%) retain substantial overlap, capturing 182 and 161 edges. Propr (63%) shows moderate agreement, incorporating 133 edges. In contrast, ESABO exhibits the lowest overlap (8%, 18 edges), likely due to its transformation of abundance data into a presence-absence format, which emphasizes significant presence/absence dynamics rather than abundance-based associations. The largest connected component (LCC) of the CN contains 110 nodes and connects 76% of occurring families. Experiments involving SPIEC-EASI’s sparsification based on association strength yielded networks with a significantly smaller LCC size in contrast to a consensus network of comparable dimensions (Supplementary Fig. S2). The final consensus network (CN) contains edges, which are validated by multiple inference methods, enhancing the ecological reliability compared to single network inference approaches. The resulting CN is considered an interaction network with potential for inferring direct trophic interactions and food web structure as well as indirect associations due to the effects of environmental filtering.

### Network modules and food web–temperature relationships

Twenty-three families are significantly correlated with temperature (δ¹⁸O NGRIP) and account for 49.8% of all reads in our data set. The consensus network exhibits a modularity score of 0.7 with temperature-correlated families defining 4 glacial and 6 interglacial modules, all being environmentally distinct. The LCC comprises six modules (Fig. 4, Supplementary Table S4), including two main interglacial modules (IG1 and IG2) and one glacial module (GLC). Other families are fragmented outside the LCC, forming small disconnected modules (Supplementary Fig. S3).

Module IG1, the largest module, consists of 27 families spanning all four trophic levels with dense linkages between mostly secondary consumers. The secondary consumers comprise diverse fish families (6 pelagic, 6 benthic), whereof seabass (Lateolabracidae) and suckerfish (Echeneidae) are linked to tertiary consumers, like small predatory whales (Monodontidae). Green algae (Chloropicaceae) are the main primary producers, linked to crustacean zooplankton (Calanidae) and planktivorous fish (Echeneidae), respectively. Module IG1 is characterized as a high trophic community (mean trophic level MTL = 2.6) including 11 families, which are positively correlated with temperature (NGRIP). The high relative abundances of fish families (Salmonidae, Sparidae, Echeneidae, Blenniidae, Lateolabracidae), diatoms (Chaetocerotaceae), green algae (Chloropicaceae) and low abundant crustacean zooplankton (Calanidae) during the Holocene support the assumption that the IG1 resembles a potential Holocene food web. Module IG2, with 9 families connected across all trophic levels, stands out with the highest abundance of tertiary consumers (MTL = 2.7). While in the IG1 primary consumers like Calanidae connect primary producers and secondary consumers, the IG2 shows direct links between green algae (Synechococcaceae, constitute 71% of the relative abundance) and herring (Clupeidae) as secondary consumers, and marine mammals. Clupeidae play a pivotal role in connecting lower trophic levels to mammalian tertiary consumers, primarily large whales (Balaenopteridae, Physeteridae, Phocoenridae). IG2 includes five families which are positively correlated with δ¹⁸O NGRIP. But the dominant families (Synechococcaceae, Mytilidae, Clupeidae, and Balaenopteridae) are more abundant during the deglacial period and partly in the Eemian, in contrast to the Holocene families of IG1.

Module GLC comprises 18 families spanning three trophic levels (MTL = 1.3). It includes 18 primary producers (mainly phototrophic protists) such as the diatoms Stephanodiscaceae, Attheyaceae, Bacillariaceae, and the haptophytes Phaeocystaceae, with the latter three containing taxa that are sea-ice associated and well-adapted to colder environments. Families of benthic and demersal consumers establish direct connections with primary producers. Notably, non-migratory wolf-fish (Anarhichadidae) are linked to Monodopsidaceae, while nematodes (Oxystominidae) form associations with haptophytes (Phaeocystaceae) and diatoms (Bacillariaceae). Module GLC represents a potential trophic community in glacial periods, evidenced by four families showing a negative correlation with temperature and their high abundance during glacial periods, including the aforementioned Bacillariaceae, Phaeocystaceae, and Attheyaceae.

Higher trophic families of module IG1 are interconnected with primary producers of module GLC by fish (Blenniidae) to diatoms (Stephanodiscaceae). Interglacial sea squirt (Cionidae) and sea otter (Mustelidae) are interconnected to glacial diatoms (Bacillariaceae). Modules IG1 and IG2 have a single connection at the highest trophic level between dolphins (Delphinidae) and small whales (Monodontidae).

### GloBI interaction types in the consensus network

Our CN approach identifies important direct trophic links that are confirmed with known trophic interactions from the GloBI database. In total 23 direct trophic links were confirmed based on 133 GloBI database entries (Supplementary Table S5). A majority (17 links) were confirmed for higher trophic interactions (between levels 3 and 4) in the deglacial-interglacial modules (Supplementary Fig. S4).

The CN exhibited a higher and more significant overlap with GloBI interactions compared to the SPIEC-EASI network, particularly at trophic level 4 (z = 2.56, *p* = 0.02 vs. SPIEC-EASI: z = 0.83, *p* = 0.19) (Supplementary Fig. S5A, right panel). Across all trophic levels, the consensus network showed a modest but positive deviation from random expectations (z = 0.7, *p* = 0.22), whereas SPIEC-EASI aligned closely with the null distribution (z = −0.89, *p* = 0.81) (Supplementary Fig. S5A, middle panel). The total overlap of edges with GloBI followed a similar trend, with the consensus network showing a stronger, though not statistically significant, agreement (z = 1.71, *p* = 0.06) compared to SPIEC-EASI (z = −0.74, *p* = 0.81) (Figure S5A, left panel).

In addition to direct trophic interactions, we examined indirect trophic interactions where edges were not directly recorded in GloBI but inferred through shared trophic connections (Supplementary Fig. S5B). While the total count of indirect edges did not significantly deviate from random expectations (CN: z = −0.26, *p* = 0.64; SE: z = −2.49, *p* = 1.0) (Figure S5B, left panel), the consensus network exhibited a significantly higher number of families within these edges (z = 4.08, *p* < 0.001) (Supplementary Fig. S5B, middle panel) and a greater sum of inferred interactions (z = 2.95, *p* < 0.001) (Figure S5B, right panel). In contrast, SPIEC-EASI alone did not show significant enrichment in either metric (z = 0.49, *p* = 0.31; z = 0.44, *p* = 0.32).

### Network flow metrics and developmental capacity

Results of the flow metric analysis are summarized in the Supplementary Table S6-S9 providing information about efficiency (A), developmental capacity (DC) and maturity (A/DC). Temperature (δ¹⁸O NGRIP) and relative sea level (RSL) are both highly correlated (*R* = 0.85, *p* = 10^− 12^, Supplementary Table S10) with the flow metrics.

**Fig. 5:**
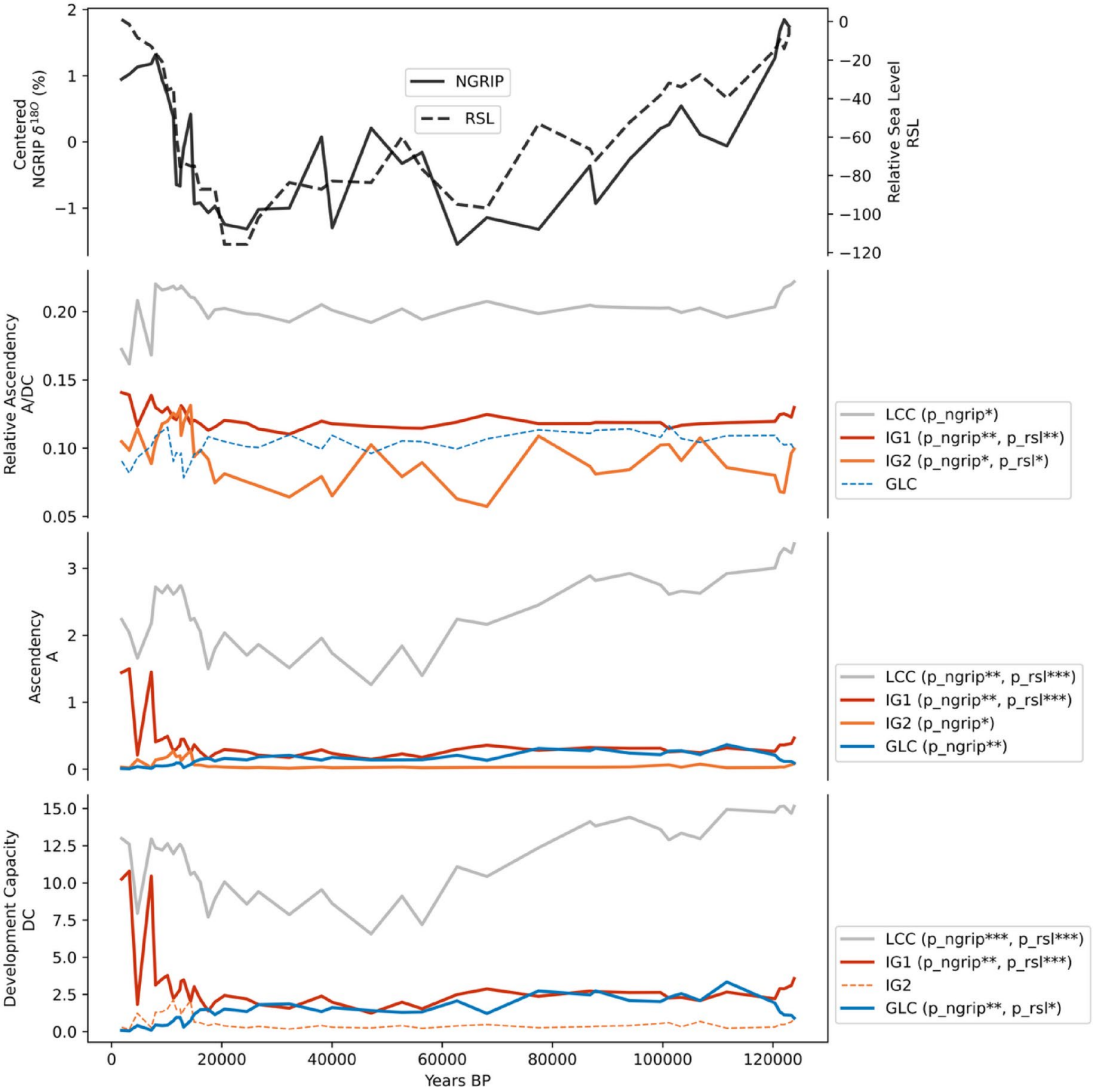
Ecosystem development increases during transitions from glacial to interglacial periods, as indicated by flow efficiency metrics. If temperature (δ¹⁸O NGRIP) or relative sea level (RSL) correlates with ecosystem (modules and LCC) flow metrics, the order of significance is indicated with * (*p*<0.05), ** (*p*<0.005) and *** (*p*<0.0005). (A/DC) Relative ascendency, a ratio between flow efficiency (ascendency A) and development capacity (DC), demonstrates how the modules IG1, IG2, and the LCC have higher maturity in warmer periods and higher relative sea level. (A) Ascendency, the metric for flow efficiency and dietary specialization, is higher in the LCC and secondary consumer module IG1 in warmer periods whereas glacial module GLC has higher ascendency in colder periods. (DC) The development capacity, representing optimal state of ecosystem development, augments in warmer periods for the LCC and IG1 but in module GLC it is higher in colder periods.

In the interglacial secondary consumer module IG1, all main development metrics correlate positively with both temperature and sea level change as shown in Fig. 5. In glacial module GLC, the flow efficiency (A) and the developmental capacity (DC) correlate negatively (*R* = −0.43) with temperature but show a lower order of significance in negative correlation of the developmental capacity (DC) to sea level change (*R* = −0.33, *p* = 0.03). Module IG2’s maturity (A/DC) correlates positively with both temperature and sea level change (*R* = 0.32 and 0.35), while the flow efficiency (A) correlates comparatively weakly and only with temperature (*R* = 0.3 and *p* = 0.048).

The development metrics of the IG1 module were k-means clustered (Supplementary Figure S6D), revealing that within IG1 high maturity (A/DC) is primarily represented by three samples in the Holocene. The maturity analysis of module IG1 (Supplementary Figure S6A-C) showed an overall increase in maturity from 0.12 to 0.14 indicating a modest rise in system organization and efficiency. Links from lower to higher trophic levels (bottom-up) increased in number (9 to 12) but decreased in their proportional contribution (15% to 8%), indicative of a dispersal of flow across more links (Supplementary Figure S6E). In contrast, links from higher to lower trophic levels (top-down) remained stable in count but increased in regulatory influence (15% to 22%), potentially due to enhanced predator specialization. Links within trophic levels (intraguild, Supplementary Figure S6E) decreased slightly in number (25 to 23) but grew in regulatory influence (34% to 38%), implying intensified interglacial competition and predation among taxa within the same trophic level.

### Robustness of network modules

The ecosystem’s robustness is evaluated by measuring the connected component size (modules IG1, IG2, GLC and the LCC) while progressively removing families and their connections, simulating taxonomic knockout (node extinction) effects. The random knockout of families establishes the general robustness of a connected component while the critical path contains families whose knockout leads to its quickest module disintegration. There may be multiple critical paths, each with varying sequences of the same deletions or even different compositions.

**Fig. 6:**
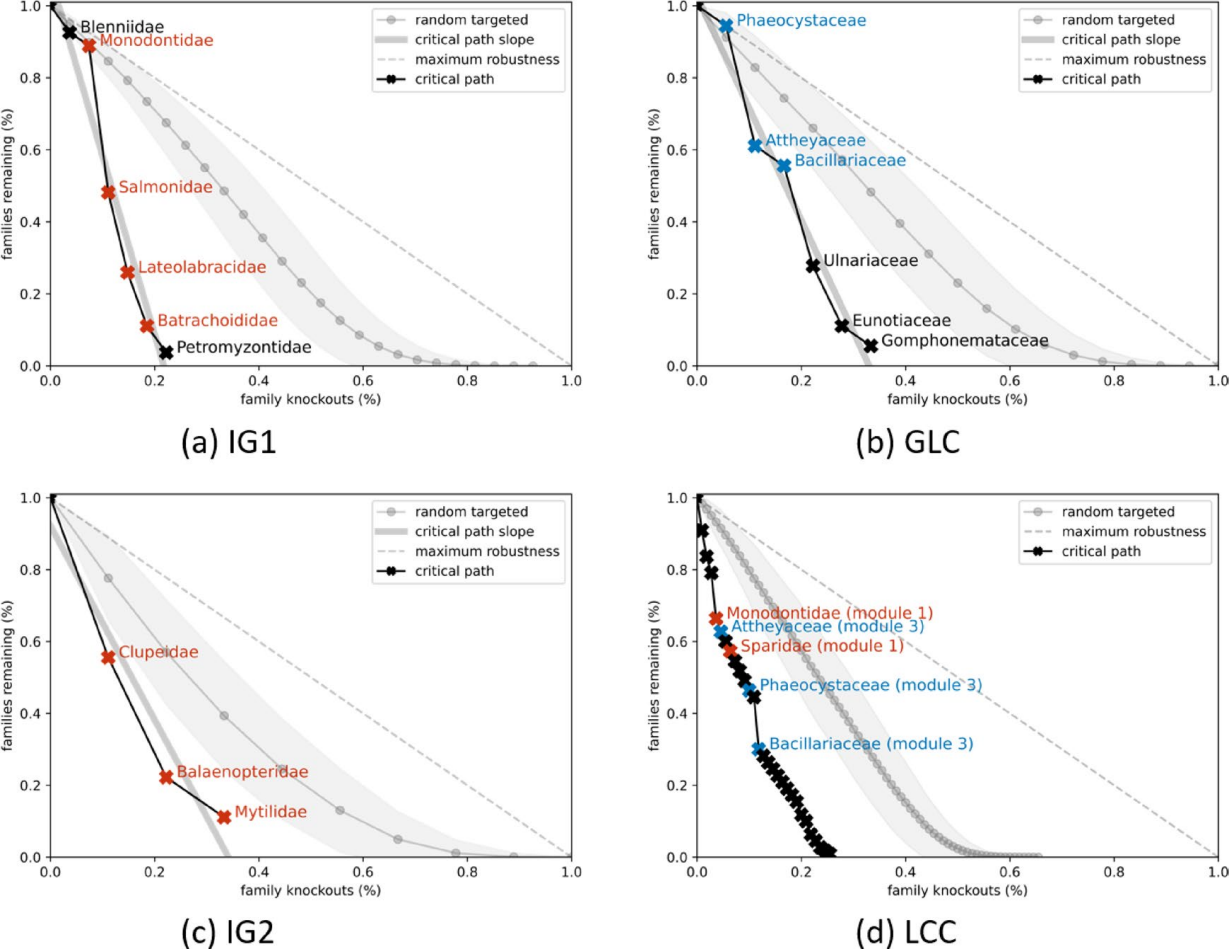
Robustness analysis of the marine ecosystem shows that glacial and interglacial modules are fragile to knockout extinction. Displayed are the mean largest connected component (LCC) size of (**a**) module IG1, (**b**) module GLC, (**c**) module IG2, and (**d**) the network’s LCC and their change due to random or targeted deletion. Randomly targeted knockouts show a clear deviation of the disintegration from maximum robustness. Critical paths illustrate the fastest disintegration of each module and network’s LCC.

Module-specific robustness analyses show distinct effects (Fig. 6). Module IG1 (secondary consumers, fish) exhibits the highest reactivity and steepest disintegration in the critical path, reaching full module disintegration after only 22% knockouts. Fish families associated with temperature heavily contribute to the loss of families along the critical path: Salmonidae 40%, Lateolabracidae 22%, and Batrachoididae 15% (Supplementary Table S11). The critical path is comparatively distant to the random deletions and maximal robustness, underscoring the susceptibility to targeted knockouts. Glacial primary production (module GLC) displays higher robustness of the critical path requiring 27% knockouts (Supplementary Table S12). Given the link density of module GLC among mainly primary producers and absence of chain-like structures, there are multiple variants of a critical path with maximum disintegration speed. But primary producers Phaeocystaceae, Attheyaceae, and Bacillariaceae – all associated with glacial conditions – are common elements occurring in the critical paths. In the highlighted critical path, the initial deletion of Phaeocystaceae is along the maximum robustness and two following deletions are close to one standard deviation of the random deletions, both indicators of higher robustness. The smaller module IG2 (tertiary consumers, mammals) requires 33% of knockouts for full disintegration (Supplementary Table S13). Knockout of two interglacial secondary and tertiary consumers – herrings (Clupeidae) and baleen whales (Balaenopteridae) – cause the main 78% of family loss. The critical path of module IG2 falls outside the standard deviation of random deletions, suggesting vulnerability to targeted perturbations; however, its smaller size constrains direct comparison with IG1.

The LCC fastest disintegration is reached after 26% knockouts with only five families along the critical path being associated with glacial or interglacial conditions (Supplementary Table S14). The single largest knockouts are by interglacial Monodontidae from module IG1 (13%) and glacial Bacillariaceae from module GLC (15%). The LCC shows high impact of targeted deletions, as the effect of knockouts along the critical path on LCC size is much stronger than for random deletions, which in turn is lower than the maximally achievable LCC size under deletion (maximum robustness).

## Discussion

Based on the taxonomic compositional changes in our sedaDNA record from the western Bering Sea, also assessed in previous studies^31,32^, we identified distinct glacial and deglacial-interglacial communities. The sedaDNA data, combined with consensus network statistics, revealed families forming potential food webs that likely were prevalent during glacial and deglacial-interglacial periods and have been influenced by climate and sea level changes. Our novel network approach provides evidence that temperature increase together with sea-level rise may have shifted different food web structures (Fig. 4) and efficiencies (Fig. 5) of the western Bering Sea in the long-term.

The presumably glacial module (GLC) consists of cold-adapted primary producers, like sea-ice algae^32^, and benthic/demersal consumers, like wolf-fish and nematodes^70^, which are characteristic for glacial conditions with low temperature, low sea level, and high abundance of sea-ice. The detected biological composition reflects a food web with strong benthic-pelagic coupling known from modern (sub)arctic ecosystems^71^, which is evident in our data by the high abundance of ice-algae (Bacillariaceae, Phaeocystaceae), benthic families, including polychaetes (Terebellidae) and nematodes (Oxystominidae). The specialized adaptation to cold environments is also evident in the increase of flow efficiency (A) in module GLC (Fig. 5) with decreasing temperature^72^. Our study shows that glacial conditions impair the food chain length of trophic interactions and affect overall food web connectance, which is detected in the low trophic diversity and strong community fragmentation of glacial modules (Fig. S3). Our findings are consistent with literature indicating that resource availability and habitat isolation have a stronger influence on large-bodied consumers, causing a decline in diversity^73,74^. The robustness analysis of glacial module GLC (Fig. 6) reveals that the glacial subsystem is more robust to perturbations because of a dense connection of primary producers. The loss of single families can potentially be compensated by other families due to similar functionality in the ecosystem. Such highly connected primary producers are likely a result of high node density^75^. To summarize, short but dense trophic connectivity, module fragmentation, and a high robustness characterize the glacial ecosystem detected in our data likely pointing to a climatically-driven bottom-up controlled food web.

During warm deglacial-interglacials marine habitat accessibility accelerated, which resulted from the submergence of shelf regions^27,76^ and reduced sea-ice. Such new ecological conditions likely offered new habitats^77^ with abundant prey for mobile secondary consumers like pelagic fish. In addition, increased horizontal fish migration^78,79^ and potential predatory control during deglacial and interglacials, likely resulted in a shift of food web structure towards higher trophic levels.

The defined interglacial modules IG1 and IG2 potentially represent two different warm periods. IG1 most likely represents the Holocene period whereas IG2 potentially represents the deglacial period, including the Bølling–Allerød, and potentially the last Interglacial, the late Eemian. Both interglacial modules (modules IG1 and IG2 in Fig. 3) are characterized by the presence of mobile secondary and tertiary consumers such as pelagic fish and whales indicating an upper trophic, horizontal (top-down) food web. The link-density among secondary consumers in module IG1 (Lateolabracidae, Blenniidae, Sparidae, Salmonidae) could point to increased predation and competition in warmer conditions during the Holocene, which in modern food webs has been shown to result from generation and dietary niche overlap^80^. Other potential top-down interactions, which could explain the connectivity of secondary consumers, are described in the literature as predatory behavior control of herbivores^81^ and non-consumptive effects on lower trophic levels such as nutrient cycling through vertical and horizontal movement^82,83^. Further, it has been assumed that warming increases small-bodied fish growth^84,85^, establishing pivotal food web components and dietary specialization opportunities for mobile predators^86^. Such specialization can result in an increase of efficient energy transfer, which is supported in our study by a positive correlation of interglacial module development with temperature (A, DC and A/DC in Fig. 5) that is derived from food web theory statistics. In particular, our results from the IG1 module show an increase in maturity and link density from lower to higher trophic levels potentially indicating a modest rise in system organization and efficiency leading to the dispersal of energy in the food web.

In contrast, herring (Clupeidae) stands out as the sole connector between primary producers and tertiary consumers in module IG2 (Fig. 3), assuming a more efficient trophic chain with lower consumer density and energy dispersal compared to IG1. While herring (Clupeidae) and baleen whales exhibit direct trophic links^87,88^, cyanobacteria and herring can be linked indirectly through increased prey quality (zooplankton) resulting in enhanced fish growth^89^. Interestingly, cyanobacteria in IG2 are comprised by marine Synechococcus and freshwater Chlorellaceae that dominate during the deglacial period (~ 11–16 kyrs BP) where rising sea-level peaked and terrestrial input fueled the Western Bering Sea^12^. Such strong perturbation could have led to compositional changes in the ecosystem^31,32^ likely resulting in enhanced herring growth^90^. A robustness analysis (Fig. 6) reveals that higher diversity of consumers and more trophic levels as exemplified in module IG1 introduce more but fragile trophic interactions. Simulated deletion of fish families such as salmon (Salmonidae), herring (Clupeidae), and top predators like small whales (Monodontidae), have strong detrimental effects on the module and ecosystem structure.

To summarize, with our analysis we suggest that higher trophic diversity, with potential trophic specialization and extensive inter-modular connectivity at the cost of increased fragility, is present in the interglacial modules pointing to a top-down regulated food web during deglacial and interglacial periods. This shift can be explained by productive spring blooms and expanded habitat availability due to the opening of the Bering Strait (~ 11 kyrs BP)^76^ and the submergence of the Siberian Shelf (~ 15–6 kyrs BP)^91^ that induced poleward migration and emergence of habitable shelf regions with ice-free conditions. In particular, module IG1 hosts warm-adapted (Blenniidae), anadromous (Salmonidae), and diadromous (Lateolabracidae) fish families, which might be attributed to poleward migration. Also, recent warming is a driver for migration by opening of marine environments^92^, likewise Holocene warming as shown to thrive migration by increased habitat accessibility^93^. Further, observational data supports that temperature implies a widespread displacement and invasion of temperature-sensitive marine species between thousands of kilometers^94–96^. The coupling between sea-ice variability and migration patterns for different marine vertebrates^97^ suggests that warmer open water conditions favor marine mammal migration^98^. Warming also triggers zooplankton and fish migration towards cooler regions^99,100^. Hence, a strong shift to fish and larger organisms in our data could indicate such migrations, which are likely driven by climate connectivity, global redistribution, and community homogenization^101,102^.

Our results for the first time apply the consensus network approach to sedaDNA shotgun sequencing data. Currently, sedaDNA shotgun sequencing is the only method that can provide time series compositional data with a large taxonomic coverage and depth allowing ecological and trophic interpretations^31,32,103^. Further, this method provides qualitative and quantitative data inferring the abundance of taxa. For now, quantitative estimates have been derived from single taxa investigations demonstrated not only for plankton^104^, but also for higher organisms, like fish^105^ and marine mammals^106^. However, there is no straightforward analysis that directly translates sedaDNA sequence reads from a mixed taxonomic composition into biomass for now, shotgun sequencing remains the only approach with reduced PCR bias and thus best representation of absolute amount of detected DNA. Evidence from recent environmental DNA suggests that metagenomic read counts (or relative abundances derived from them) can serve as a proxy for biomass for some taxa groups like plankton^104,107^ and invertebrates^108^. However, for higher animals, variations in genome and body size alter the relationship between read count and biomass. Further, the sedaDNA quality and quantity is influenced by environmental factors (temperature, oxygen level, water chemistry, and the source distance^109^ during deposition and is further modified after burial. For example, mineral composition of the sediment determines the binding of extracellular DNA fragments and sediment particles for long-term preservation of DNA^110^. How taphonomic issues vary between different taxonomic groups is largely unknown, but they influence the amount of DNA buried and require more complex analysis for biomass estimates. Further, post-mortem DNA fragmentation and modification (post mortem damage patterns) is a result of aging DNA in the sediments^111^, but the detection of these patterns provide evidence for authenticity of DNA (Supplementary Fig. S7). The fragmented nature of DNA and post-mortem modifications are present properties of the underlying sedaDNA dataset, but have only a limited effect on the compositional data. This is supported by the continuous presence (with varying relative abundance) of most of the families in our sediment core and the consistent compositional trends at least for the last 20,000 years BP in our data and in a second sedaDNA dataset from a geographically close locality^28^. However, due to incomplete reference databases^112^ the representation of all taxa involved in trophic interactions might be limited to popular and abundant taxa in recent sub-arctic marine food webs. For now, our study focuses mostly on eukaryotic organisms and pelagic interactions and did not implement microbial groups (except for Cyanobacteria). Integrating microbes would target important ecosystem processes such as microbial cycling^113^(transfer of carbon and nutrients to higher trophic levels) and trans-kingdom (e.g. microbe-host) interactions^114^ which are highly relevant for ecosystem productivity and food web structure. However, the network implementation remains challenging^115^ due to the high diversity, rapid turnover rates, and complex, often indirect interaction pathways of microbial communities.

The method of the consensus network originates from gene regulatory networks^42^, but in this study we show how the underlying principle of “crowd knowledge"^112^ and merging different interaction types^116^ is applicable and beneficial to ecological networks. We validate the consensus network approach using synthetic data obtained from a generalized Lotka-Volterra model (Fig. 3). While the simulations confirm a reduction in false discoveries (FP), the synthetic data primarily serves as a method validation and does not fully capture the complexity of real food webs, especially compounded with environmental factors. The verification of the consensus network with known trophic interactions from GloBI, particularly at higher trophic levels containing predator-prey interactions, supports its ecological reliability. This highlights that although co-occurrences alone do not indicate direct trophic links, incorporating them into a consensus approach leverages their studied ability to reflect trophic hierarchies across varying environmental conditions^117^. However, the consensus approach inherently functions by an edge reduction mechanism, prioritizing precision over completeness and capturing only a fraction of the potentially strongest interactions. As a result, subtle ecological dynamics and weak or transient interactions with many edges may remain undetected^118^. This is evident from the presence of many indirect family interactions in the CN and its low coverage of direct links compared to the GloBi database. Additionally, given the millennial-scale dynamics of our study, the GloBI database may be incomplete in this context, meaning our network could potentially also highlight previously undiscovered ecological interactions and long-term trophic dynamics. In summary, verification of the results shows that treating each family-family connection as a significant, bidirectional relationship enables the application of relevant food web methods and interpretations over millennial timescales.

Despite the challenges of sedaDNA data and limitations in precision and interpretability of ecology networks, our study pioneers the reconstruction of a food web through shotgun-sequenced sedaDNA, combined with validation through simulations, to link trophic communities and food web control to long-term climate and environmental dynamics. Our approach offers an advanced network analysis on paleoecological data to infer mostly pelagic ecosystem composition and food web structure across millennial times based on sedimentary ancient DNA. Assuming previous warming periods can serve as a surrogate for the current temperature rise, the understanding of distinct patterns of glacial, deglacial and interglacial food webs in the past is crucial for assessing future marine ecosystem functionality and decision making. Observational and experimental studies provide controversial perspectives on marine food web development under enhanced temperature rise. It has been shown that warming can initially enhance primary production and resource availability, increasing energy input into marine food webs^119,120^, but also increase metabolic demand, reducing trophic transfer efficiency and weakening energy flow to higher trophic levels. Further, warming triggers poleward range expansions of mobile taxa such as fish and marine mammals (e.g., northward shifts of boreal species) into newly accessible habitats restructuring predator–prey interactions with intensified top-down pressure^121,122^. Summarily, the future trajectory of marine food webs under continued warming remains difficult to predict, as species-specific adaptation, plasticity, and the emergence of novel interactions cannot yet be robustly quantified or integrated into long-term ecosystem forecasts.

## Supplementary Information

Below is the link to the electronic supplementary material.

Supplementary Material 1

Supplementary Material 2

Supplementary Material 3

Supplementary Material 4

Supplementary Material 5

Supplementary Material 6

Supplementary Material 7

Supplementary Material 8

Supplementary Material 9

Supplementary Material 10

Supplementary Material 11

Supplementary Material 12

Supplementary Material 13

## Data Availability

The raw shotgun DNA data is publicly available under the BioProject accession PRJEB66300 on the European Nucleotide Archive (ENA). The data and analysis code are publicly available under https://doi.org/10.5281/zenodo.20796956. The code for the simulation analysis is publicly available under https://doi.org/10.5281/zenodo.20797564.
