# Supplementary material for: Past subarctic marine food web shifts recovered by sedaDNA and network analysis
Source: Sci Rep. 2026 Jul 12;16:21648. doi: 10.1038/s41598-026-60317-z (PMC13357734; doi:10.1038/s41598-026-60317-z)
Supplement: Supplementary file 1 — Supplementary Material 1 [file 41598_2026_60317_MOESM1_ESM.docx]

# Extended Material and Methods

## Network Inference methods

For the purpose of network inference of ecological systems, we selected a diverse set of methods—SPIEC-EASI, Spearman, Propr, ESABO, SparCC, CCREPE, and EcoCopula—that are novel or frequently employed in ecological network analysis. These methods represent a selection from state-of-the-art tools and were chosen to encompass a methodological range of both direct and indirect interactions (Table S1).

**Table S1:** Overview of the used inference methods

| **Inference Method** | **Inference Name & Description** | **Resulting Link Type** |
| --- | --- | --- |
| **Spearman** | Pairwise Pearson correlation of abundance rank values. Sensitive to indirect associations. | Co-Occurrence |
| **SPIEC-EASI** | Conditional dependence identified by inverse covariance matrix, inferred with graphical model. Suitable for compositional data. | Direct association |
| **ESABO** | Significant entropy shift after boolean operation on presence-absence data. Required data transformation may disregard continuous ecological dynamics. | Co-Occurrence |
| **Propr** | Proportionality of the log-ratio variance. Assumes a constant proportional relationship between families across samples. | Co-Occurrence |
| **SparCC** | Pearson correlation from log-ratio variance, assuming that most family pairs are uncorrelated. | Co-Occurrence |
| **CCREPE** | Significant ensemble score from four  correlation metrics (two each: similarity &  dissimilarity). Limited in resolving direct and indirect interactions. Requires computationally intensive permutations. | Co-Occurrence |
| **EcoCopula** | Copula ordination with optional environmental covariates, inferred with a graphical model. Not explicitly designed for compositional data. Computationally demanding for large networks. | Direct association |

## NGRIP and IP25 as an Indicators of Past Climate Variability

The application of stable oxygen isotopes retrieved during the North Greenland Ice Core Project (δ¹⁸O NGRIP) as a proxy for the paleo-climate conditions in the northern hemisphere is supported by a multi-proxy study in the Mediterranean Sea that identified periodicities (8000, 5000, 3300, 1470 years) matching those in the Greenland ice cores, demonstrating a strong link between marine and atmospheric processes. For past temperature the δ¹⁸O NGRIP dataset^56^ has been used where age data points are selected closest to the sample ages. The extracted NGRIP δ¹⁸O-isotope values are centered and standardized by subtracting their mean value and division by standard deviation. This results in the δ¹⁸O fluctuations between glacial and interglacial conditions and for simplicity it is called temperature in this study. Fig. S1 further reinforces this connection, showing a significant negative correlation between δ¹⁸O NGRIP and IP_25_, a sea-ice proxy, from two cores (77KL, the core of this study, and 12KL, a neighboring core). This confirms that δ¹⁸O NGRIP reflects broad climatic trends in the northern hemisphere, making it a suitable paleo-climate proxy, utilized for studying marine ecosystems responses to long-term global climatic changes.

## Interaction Types in the Consensus Network

Direct interactions from GloBI were queried for each family, retaining only those between families included in our list (Table S14). Erroneous results, such as interactions involving incorrect species, were excluded. The list was further refined to include only unidirectional, trophic interactions (“eats”, “preysOn”). Each result was filtered to ensure uniqueness, particularly in terms of the "study_title" (interaction reference) field. Links in the consensus network were classified as "direct" (trophically validated by GloBI) if at least one interaction reference was present. The number of unique references served as a measure of validation strength, determining the weight of each edge (Fig. S4). Indirect interactions were identified by retaining only the links in the consensus network not present in GloBI. For each remaining (non-direct) interaction, the associated families were queried in GloBI to obtain two lists of interacting organisms. The intersection of these lists identified common neighbors between the linked families. The presence of at least one common trophic neighbor defined an indirect interaction, with the number of common neighbors determining the edge weight. The sum of unique references for all interactions within each neighbor quantified the indirect interactions (Fig. S4).

## Development metrics of module IG1

The temporal development metrics (A, DC, and A/DC) of module IG1 were clustered into two groups using k-means (Fig. S6F). Samples aged 1820, 3220, and 7210 were assigned to cluster 1, characterized by higher maturity values (≥0.14), while cluster 0 contained samples with lower maturity values. A comparative analysis between the two clusters was performed, highlighting only the differences (increases and decreases) in cluster edges.

To assess individual edges, maturity values for samples in each cluster were calculated separately for each directed edge, yielding the maturity difference between clusters. If an edge exhibited a greater maturity increase in cluster 1, its maturity difference contributed to the total increase in maturity (black edges in Fig. S6A-C). Conversely, if an edge had higher maturity in cluster 0, it contributed to the maturity delta of cluster 0 (gray edges in Fig. S6A-C). The total maturity increases in cluster 1 was determined as the sum of all edges with a higher maturity difference in this cluster. The proportional contribution of each edge was calculated as its maturity difference relative to the total increase.

Changes were further categorized into regulatory influence by edge direction: bottom-up (lower to higher trophic level), top-down (higher to lower trophic level), and intraguild (within the same trophic level). This classification allowed for identifying where structural shifts occurred. Fig. S6D illustrates the proportional changes in regulatory control between clusters.

## Damage pattern analyses

The investigation of damage patterns for three selected families was conducted using MapDamage v. 2.0.8[1](https://www.zotero.org/google-docs/?yvNd47). Prior to the analyses, we grouped the samples into six subgroups by age (group 1: 1.8–11.2 ka, group 2: 11.8–18.8 ka, group 3: 20.5–47.1 ka, group 4: 52.7–87.8 ka, group 5: 94.0–111.2 ka, group 6: 120.4–123.9 ka) by merging the raw sequencing data of the according samples, and repeated the bioinformatic analysis. In short, raw sequencing data was quality checked with Fastqc, deduplicated with clumpify, trimmed and merged with fastp, and taxonomically classified with kraken2 using a confidence threshold of 0.2 against the nt database (release April 2021)[2](https://www.zotero.org/google-docs/?USiiIJ). After that, reads classified to three families (Bathycoccaceae, Salmonidae, Balaenopteridae) were extracted from the dataset and mapped against the RefSeq references (Bathycoccus prasinos, GCF_002220235.1; Onchorynchus kisutch, GCF_002021735.2; Balaenoptera musculus, GCF_009873245.2) downloaded from NCBI, April 2024. Then, MapDamage with the options ‘rescale’ and ‘single-stranded’ was applied to estimate the post mortem nucleotide substitutions in order to verify the ancientness of the DNA molecules. The frequency of C to T changes, diagnostic changes in the read base-pair composition due to deamination processes acting on the ancient DNA molecules, is detected. The expected increase of C to T changes towards the ends of the ancient DNA molecules reflects post mortem damage of the molecules and indicates the authenticity of the sedimentary ancient DNA data acquired from the sediment core SO201-2-77KL (Fig. S7).

# Supplementary Figures


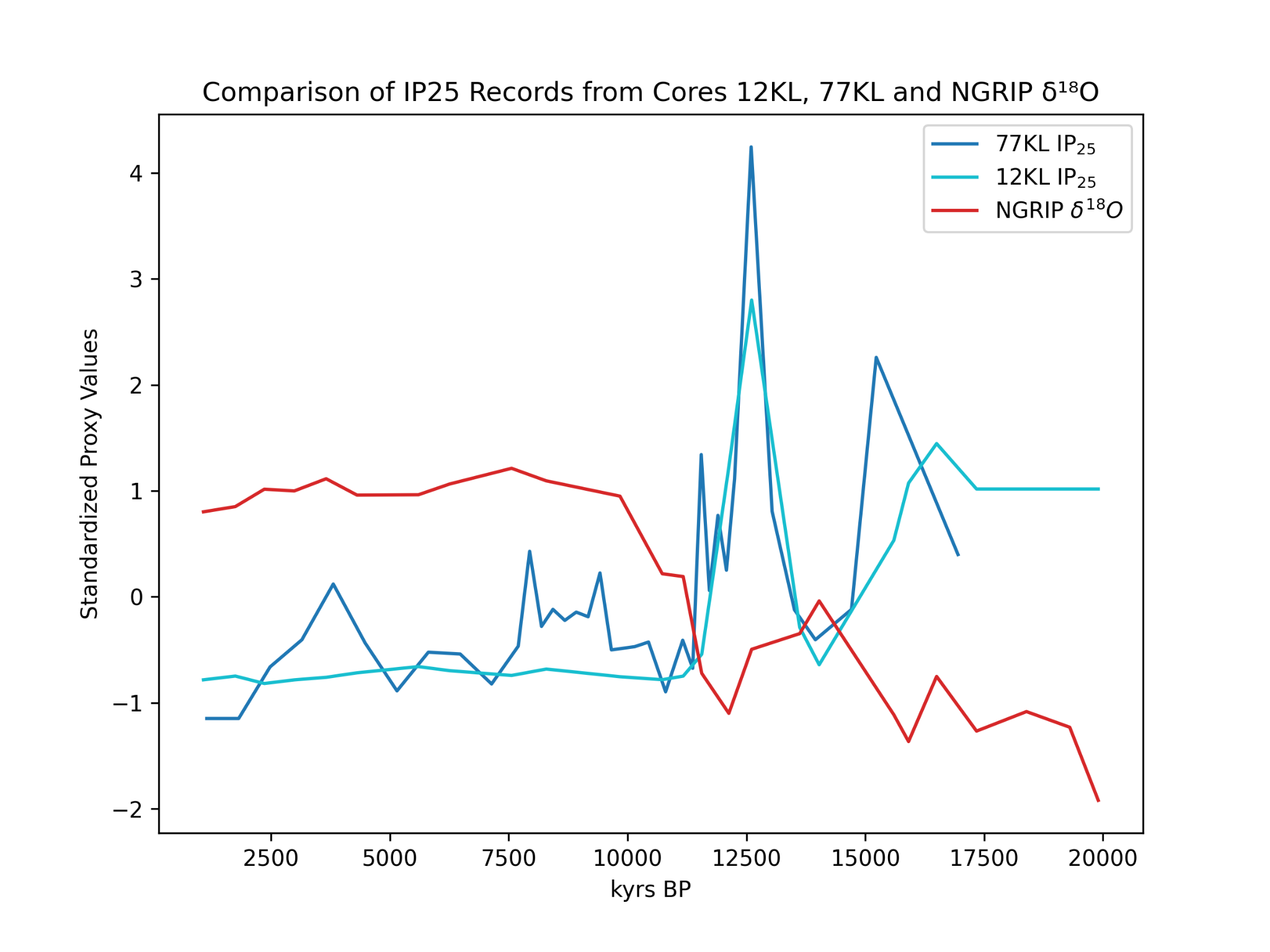


**Figure S1 | Standardized IP25 records from cores 12KL (dark blue) and 77KL (teal) plotted alongside standardized NGRIP δ¹⁸O (dark red) to assess their correlation.** Pearson and Spearman correlation coefficients indicate a significant negative relationship between IP_25_ and δ¹⁸O NGRIP. 12KL shows a Pearson correlation of r = -0.57 (p = 0.0003) and a Spearman correlation of rₛ = -0.75 (p = 1.7 × 10⁻⁵), while 77KL shows a Pearson correlation of r = -0.78 (p = 5 × 10⁻⁶) and a Spearman correlation of rₛ = -0.50 (p = 0.002). These results suggest that increases in IP_25_, a sea-ice proxy, correspond to lower δ¹⁸O NGRIP values, consistent with colder conditions in the North Atlantic region.

## **
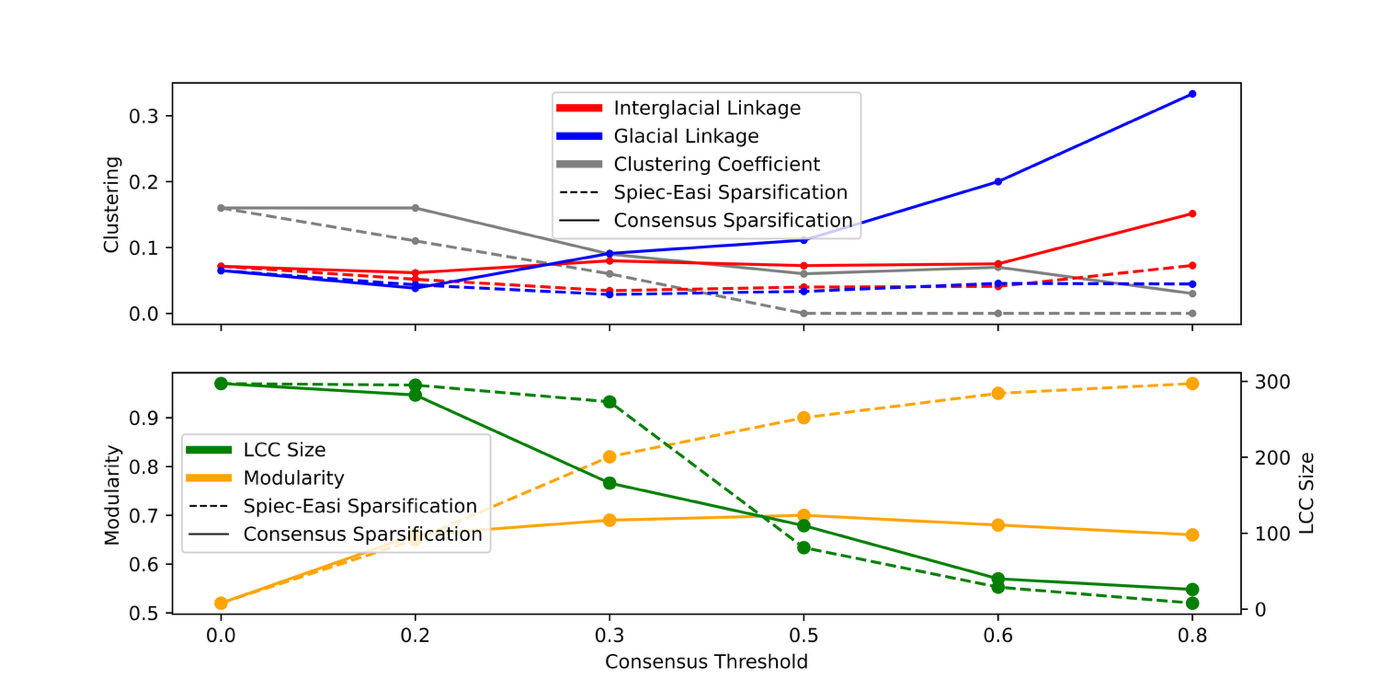
**

**Figure S2 | The method of sparsification by consensus yields larger Largest Connected Component (LCC) sizes and promotes higher environmental clustering in comparison to sparsification based solely on the association strength of the base network (SPIEC-EASI).** As the threshold increases, the modules within the consensus network become increasingly distinguishable in terms of their climatic linkage and consequently glacial/interglacial characteristics. This implies that glacial and interglacial taxa are more likely to be interconnected, representing a notable advantage of the consensus methodology. However, as the consensus threshold rises, the rate of modularity growth is comparatively slower than that observed in base network sparsification. When modularity surpasses a threshold of 0.8, it results in a band topology and module fragmentation, leading to a loss in the informational integrity of the network. The second distinct advantage of the consensus methodology lies in its ability to maintain a significant LCC size while fostering clustered modules.

**
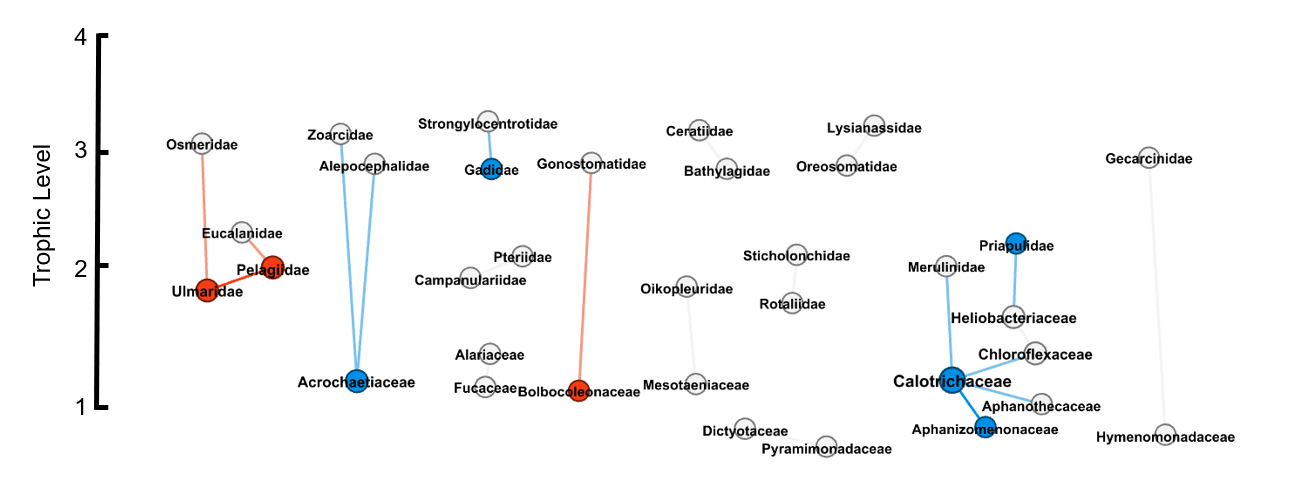
Figure S3 | The modules outside the Largest Connected Component (LCC) depict a fragmented, isolated ecosystem.** Node size represents the node degree centrality. Red and blue nodes show positive and negative Spearman correlations with temperature. Among these fragments, there are two interglacial and three glacial modules. Three glacial modules exist outside and only one within the LCC, suggesting a higher degree of fragmentation in the glacial ecosystem.


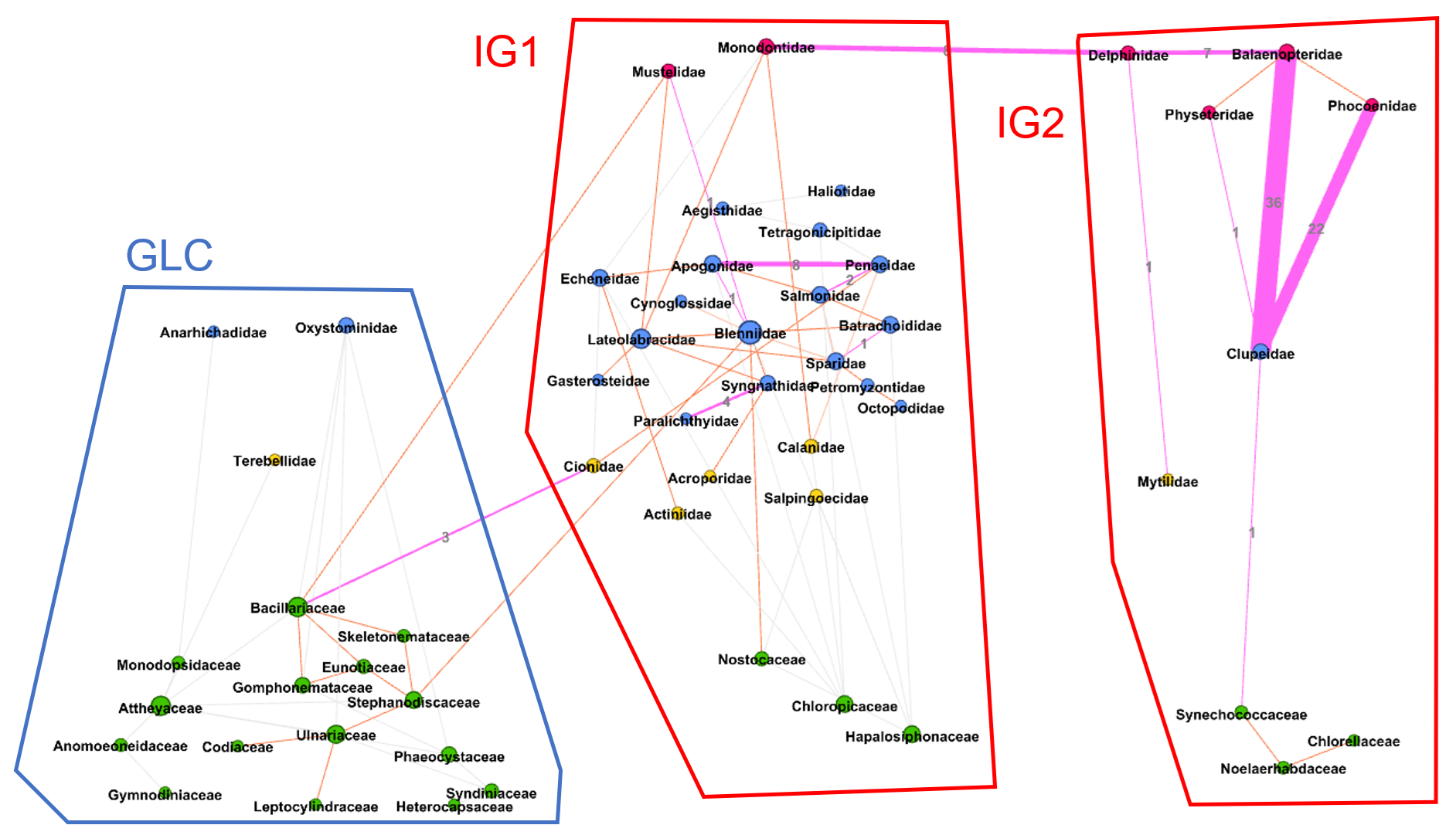


**Figure S4 | Consensus network overlap with the Global Biotic Interactions (GloBi) database illustrates evidence of trophic interactions in Interglacial Modules IG1 and IG2.** The figure highlights 16 trophic edges of the consensus network which are overlapping with GloBi (pink). Edge labels indicate the number of unique interaction references. Edges with indirect interactions (orange) in the consensus network represent links between families that lack a direct trophic connection in GloBi but share common neighboring families, suggesting potential trophic dependencies between them. The interglacial modules IG1 and IG2 are characterized by a higher diversity of direct and indirect trophic interactions, with "preysOn" or "eats" associations predominantly occurring at trophic levels 3 and 4. In IG1, the dense cluster of fish exhibits heightened trophic connectivity, while IG2 shows almost complete trophic referencing at higher levels, with Clupeidae playing a central role. The concentration of "preysOn" and "eats" interactions in these modules suggests a top-down, interaction based regulatory effect. In contrast, the glacial module GLC lacks trophic interactions, illustrating a bottom-up, environmentally driven ecosystem structure.


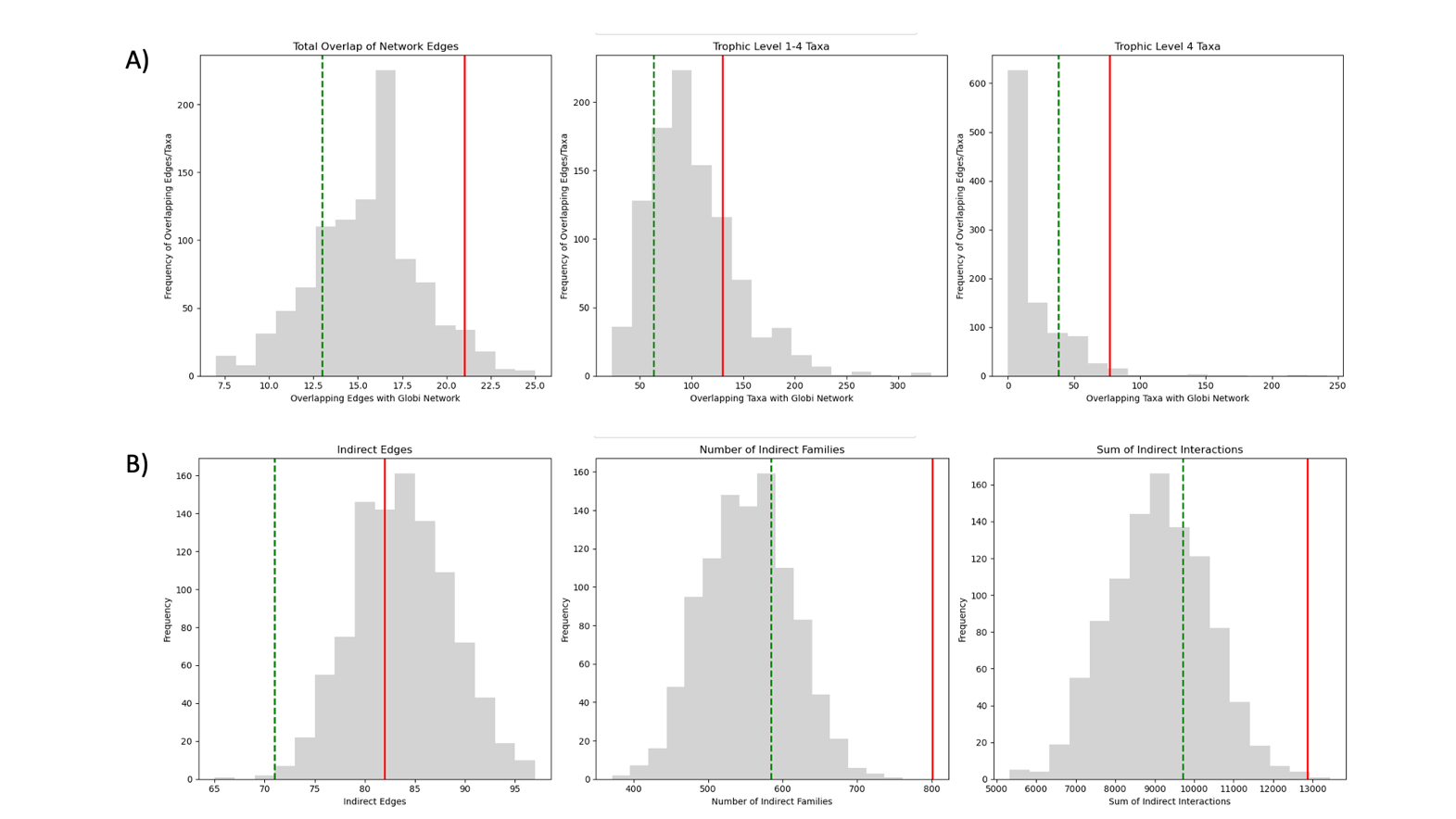


**Figure S5 | Overlap of Consensus and SPIEC-EASI Networks with GloBI Trophic Interactions.** Histograms compare network overlap with the GloBI database against a null distribution generated from 1,000 randomized networks. Vertical lines indicate the actual overlap of the consensus network (CN, red) and SPIEC-EASI (SE, green) networks with GloBI. **(A)** Direct interactions as edges in the CN which also occur in GloBI. Left panel: total edge overlap (left panel, CN: z = 1.8, p = 0.06; SE: z = -0.78, p = 0.83), middle panel: amount of overlapping taxa across all trophic levels (CN: z = 0.68, p = 0.21; SE: z = -0.9, p = 0.83), and right panel: amount of taxa in trophic level 4 (CN: z = 2.27, p = 0.02; SE: z = 0.73, p = 0.18). **(B)** Indirect interactions as CN edges which do not occur in GloBI but share the same neighbours in GloBI. Left panel: amount of indirect CN edges. Middle panel: number of indirect families involved in each indirect edge. Right panel: number of interactions involved in each indirect edge.


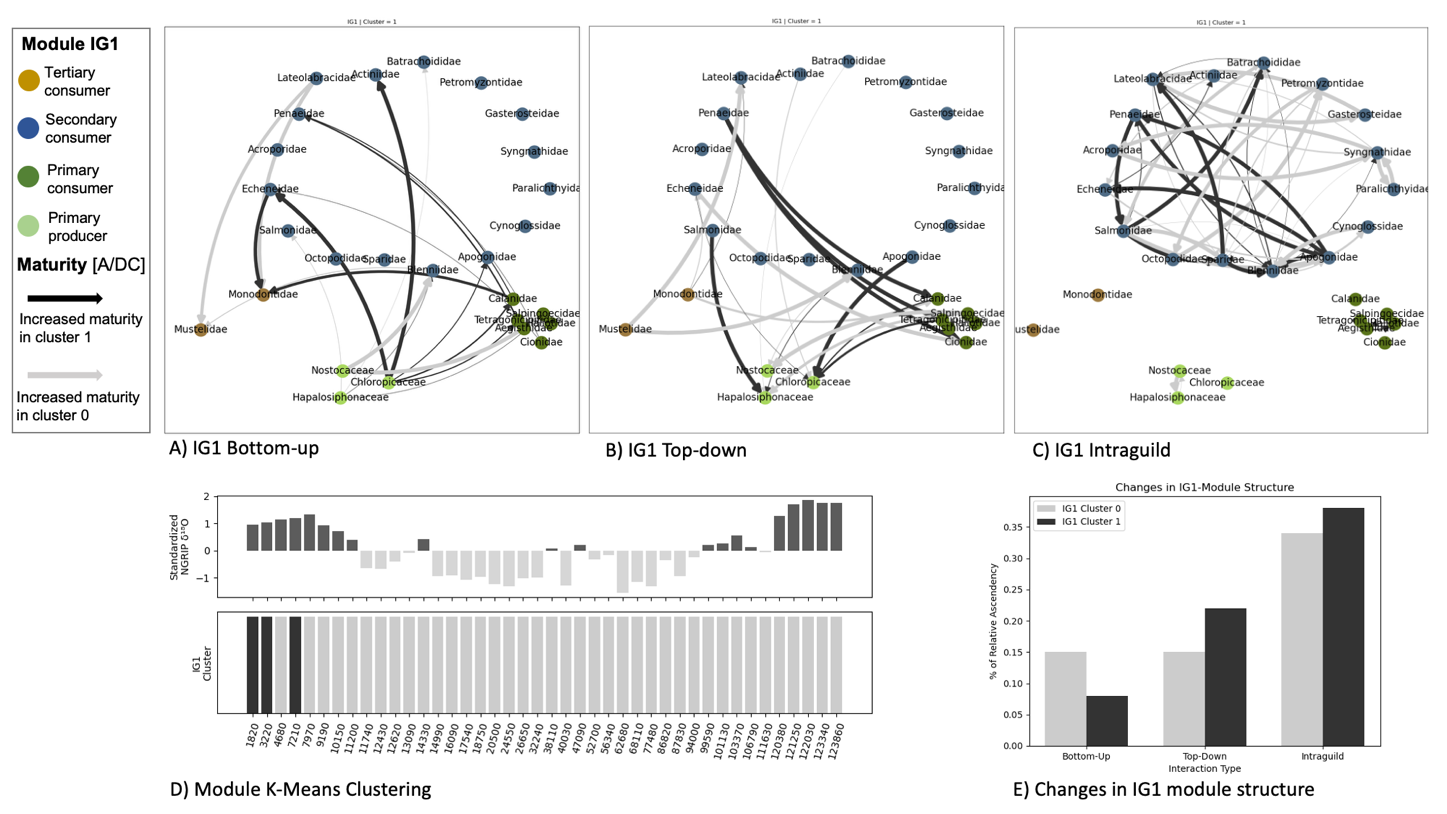


**Figure S6 | Development metrics of module IG1.** (A-C) IG1 module with directed edges showing increased (black) and decreased (gray) relative ascendency in cluster 1 compared to cluster 0. (A) Bottom-Up interactions (source trophic level lower than target), (B) top-down interactions (source trophic level higher than target), and (C) intraguild interactions (identical source and target trophic levels). (D) δ¹⁸O NGRIP variation and k-means clustering of IG1 development metrics (A, DC, A/DC) into high (cluster 1) and low (cluster 0) values. (E) Bar charts of regulatory influence, illustrating the proportional distribution of relative ascendency changes across interaction types. The results of IG1 indicate that, in cluster 1, the balanced top-down and bottom-up control observed in cluster 0 shifts, with top-down interactions strengthening while bottom-up influence declines relative to cluster 0.


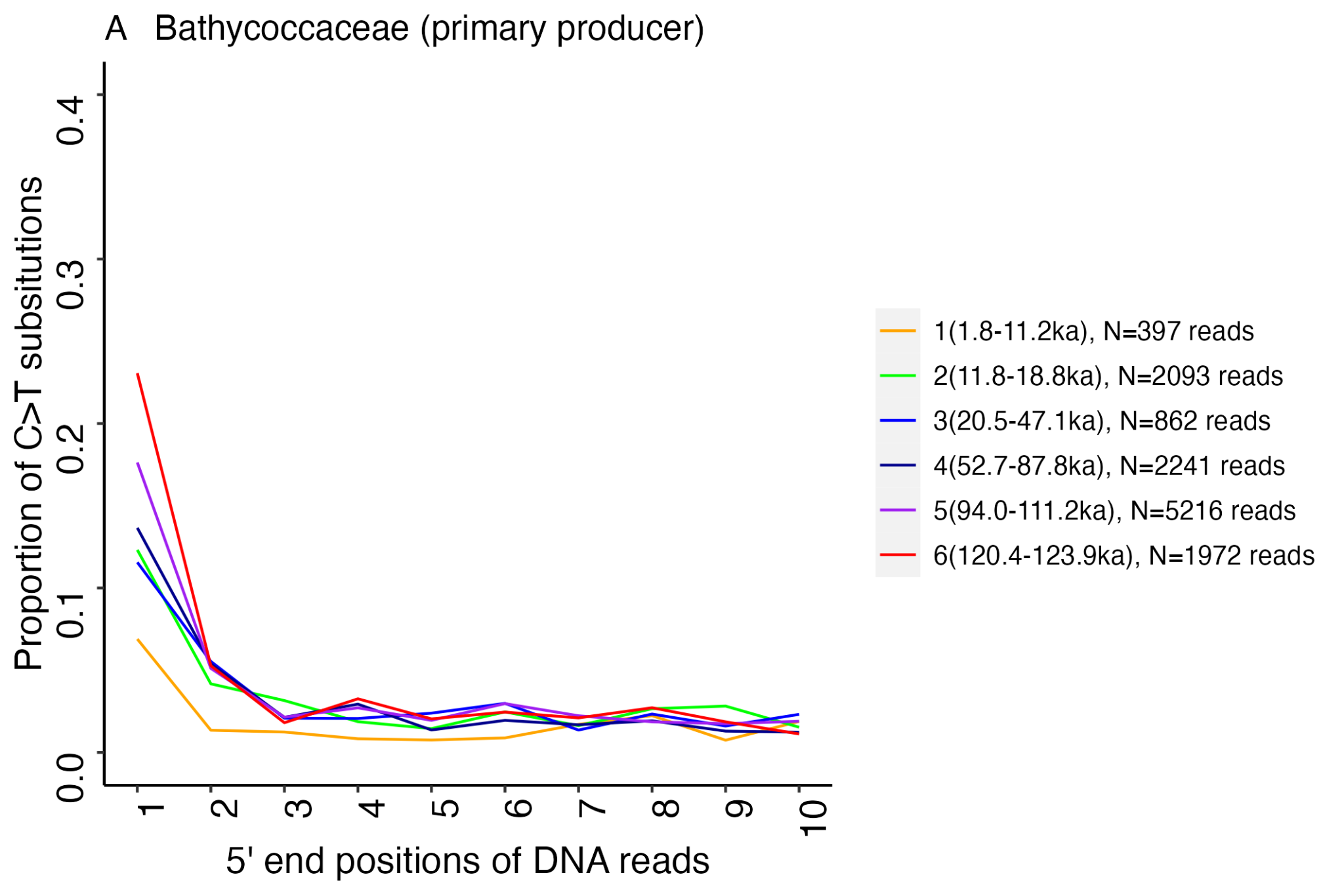


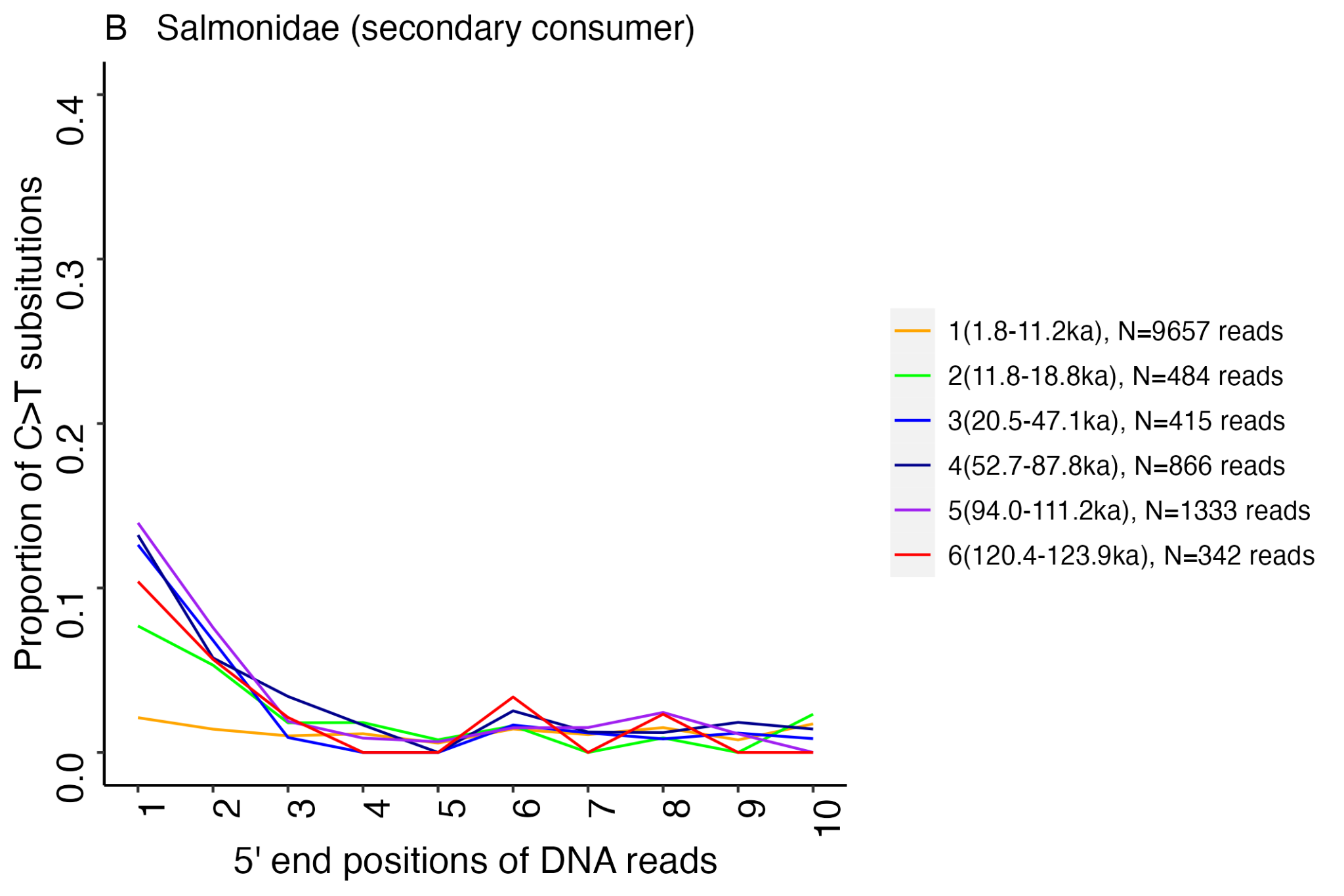


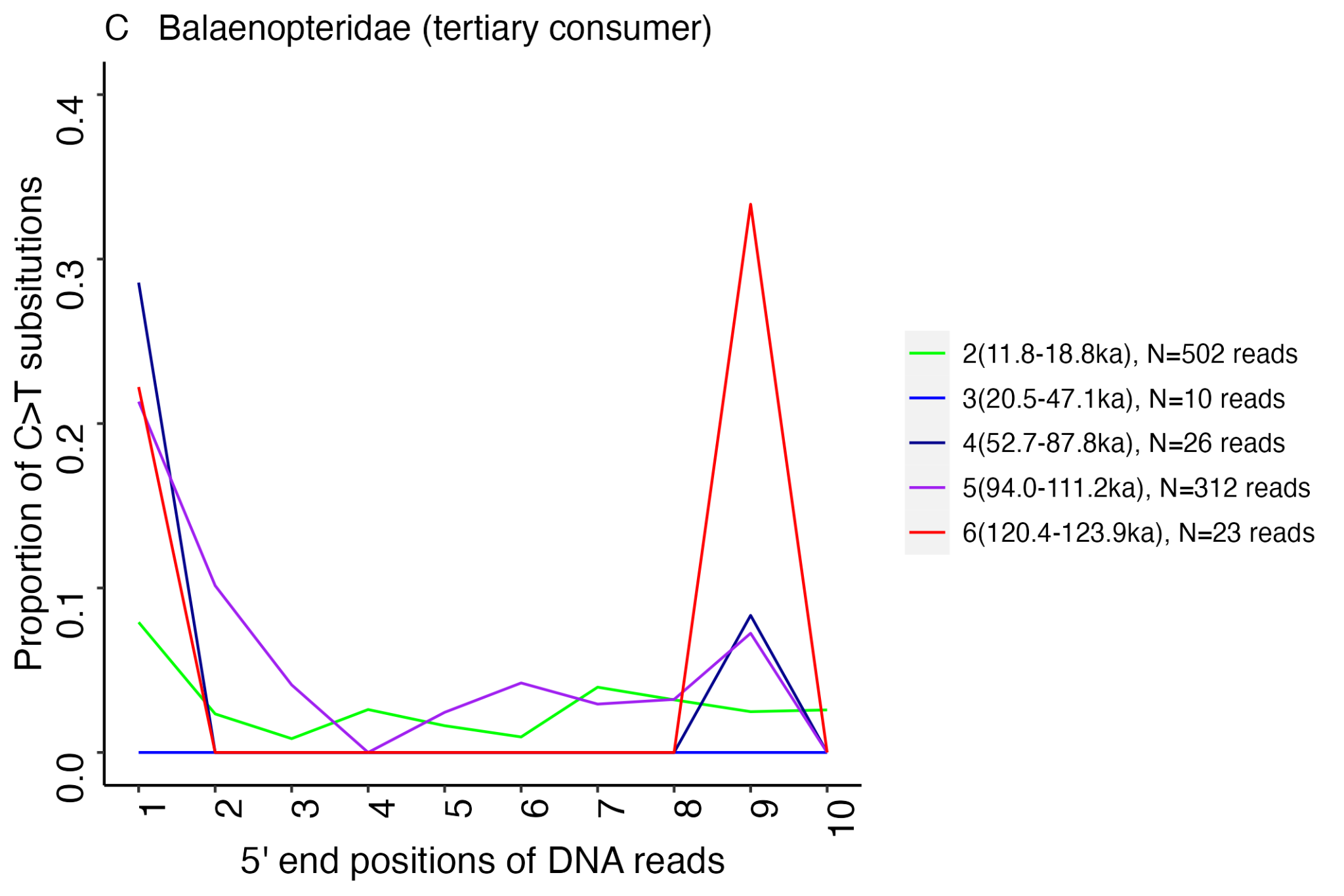


**Figure S7 | C to T substitution frequency of the last 10 base pairs** for reads taxonomically assigned to the families: Bathycoccaceae (A), Salmonidae (B), and Balaenopteridae (C). The colors indicate the age groups. The read numbers used for the MapDamage analysis are given as N.

**Table S1**| Overview of the used inference methods.

**Table S2** | Families with their taxonomic groups and trophic level.

**Table S3** | Results from the PubMed Search for all families.

**Table S4** | Families of the LCC with their taxonomic group, trophic level and correlation with temperature.

**Table S5** | List of trophic links in the consensus network with GloBI references.

**Table S6** | Flow metrics of module IG1 at different years Before Present (*BP*), including ascendency (*A*), average mutual information (*AMI*), total system throughput (*TST*), complexity (*H*), development capacity (*DC*), and the relative ascendency as a ratio of *A* to *DC* as (*A/DC*).

**Table S7** | Flow metrics of module GLC at different years Before Present (*BP*), including ascendency (*A*), average mutual information (*AMI*), total system throughput (*TST*), complexity (*H*), development capacity (*DC*), and the relative ascendency as a ratio of *A* to *DC* as (*A/DC*).

**Table S8** | Flow metrics of module IG2 at different years Before Present (*BP*), including ascendency (*A*), average mutual information (*AMI*), total system throughput (*TST*), complexity (*H*), development capacity (*DC*), and the relative ascendency as a ratio of *A* to *DC* as (*A/DC*).

**Table S9** | Flow metrics of the largest connected component (LCC) at different years Before Present (*BP*), including ascendency (*A*), average mutual information (*AMI*), total system throughput (*TST*), complexity (*H*), development capacity (*DC*), and the relative ascendency as a ratio of *A* to *DC* as (*A/DC*).

**Table S10** | Correlation coefficients (R) and p-values (p) for the relationship between various flow metrics (*A/DC, A, DC*) across different modules (IG1, IG2, GLC) and the LCC with NGRIP and relative sea level (RSL) data.

**Table S11** | Results of the knockout extinction analysis for module IG1 showing the proportion of knockouts, the family going extinct, the remaining proportion of families, and the associated color coding.

**Table S12** | Results of the knockout extinction analysis for module GLC showing the proportion of knockouts, the family going extinct, the remaining proportion of families, and the associated color coding.

**Table S13** | Results of the knockout extinction analysis for module IG2 showing the proportion of knockouts, the family going extinct, the remaining proportion of families, and the associated color coding.

**Table S14** | Results of the knockout extinction analysis for the LCC showing the proportion of knockouts, the family going extinct, the remaining proportion of families, and the associated color coding.
